# MCP-enabled LLM for meta-optics inverse design: leveraging differentiable solver without LLM expertise

**DOI:** 10.1515/nanoph-2025-0507

**Published:** 2025-12-04

**Authors:** Yi Huang, Bowen Zheng, Yunxi Dong, Hong Tang, Huan Zhao, Rakibul Hasan Shawon, Sensong An, Hualiang Zhang

**Affiliations:** Department of Electrical and Computer Engineering, University of Massachusetts Lowell, Lowell, USA; Department of Electrical Engineering, University of North Texas, Denton, USA

**Keywords:** TorchRDIT, inverse design, large language model (LLM), model context protocol (MCP), automatic differentiation

## Abstract

Automatic differentiation (AD) enables powerful metasurface inverse design but requires extensive theoretical and programming expertise. We present a Model Context Protocol (MCP) assisted framework that allows researchers to conduct inverse design with differentiable solvers through large language models (LLMs). Since LLMs inherently lack knowledge of specialized solvers, our proposed solution provides dynamic access to verified code templates and comprehensive documentation through dedicated servers. The LLM autonomously accesses these resources to generate complete inverse design codes without prescribed coordination rules. Evaluation on the Huygens meta-atom design task with the differentiable TorchRDIT solver shows that while both natural language and structured prompting strategies achieve high success rates, structured prompting significantly outperforms in design quality, workflow efficiency, computational cost, and error reduction. The minimalist server design, using only 5 APIs, demonstrates how MCP makes sophisticated computational tools accessible to researchers without programming expertise, offering a generalizable integration solution for other scientific tasks.

## 1 Introduction

As the demand for compact, high-performance optical devices with diverse functionalities increases, metasurfaces, offering unprecedented manipulation of electromagnetic (EM) waves within an ultra-low-profile scale, emerge as the definitive solution for applications across sensing, imaging, and telecommunications [1], [2], [3]. Automatic differentiation (AD) based inverse design methodologies have shown promising capabilities of designing high-performance metasurfaces efficiently, with the feature of seamlessly integrating with machine learning (ML) frameworks [4], [5], [6], [7], [8], [9], [10], [11], [12], [13]. ML inverse design has delivered fast surrogates and flexible mappings for nanophotonics, but it is data-driven: model accuracy and generalization depend on the coverage and quality of training sets. Representative work spans supervised predictors and tandem or physics-driven architectures that mitigate non-uniqueness, as well as deep generative models that handle one-to-many inverse mappings probabilistically [14], [15], [16], [17], [18]. These approaches enable rapid exploration yet rely on curated datasets and priors shaped by training distributions. By contrast, AD directly couples a Maxwell solver to gradient-based optimization. The optimization is data-free and deterministic at the algorithmic level: given the same physical model, loss, and initialization, the gradients and updates are fixed, so performance scales with solver fidelity rather than dataset size. This distinction motivates an AD-centric route for reliable metasurface inverse design and positions data-driven methods as complementary. Nevertheless, these existing tools tend to address only specific problem domains. To further push the implementation of these tools for more complex applications, such as large-scale design optimization or multiphysics simulations, researchers face considerable technical challenges. They must combine in-depth theoretical knowledge of the relevant physical mathematics with advanced software engineering implementations. Consequently, the steep learning curve and specialized skill barriers often restrict the widespread adoption of these tools in a broader research context.

Meanwhile, recent advances in large language models (LLMs) infrastructures and applications have served as a catalyst for revolutionary changes across industrial sectors [19], [20], [21]. This explosive growth of LLMs has been rapidly transforming the optical design, with the recent breakthroughs including the demonstration of direct applications of LLMs and transformer models to the design of nanophotonics [22], [23], [24], [25], and agentic generative LLMs frameworks for high efficiency autonomous multi-objective inverse design [26]. However, the direct implementations of LLMs face a fundamental limitation: they essentially perform statistical inference and pattern matching rather than physical computations. While LLMs excel at text generation, content summarization, and productive task automation with agentic frameworks [27], [28], which may achieve a certain level of accuracy in specialized design tasks with well-designed frameworks and sufficient pre-trained datasets, they still lack strict enforcement of Maxwell’s equations and other mathematical and physical constraints. Instead of viewing LLMs and numerical inverse design methodologies as competing approaches, we propose a complementary paradigm: LLMs serves as an orchestration layer that preserves the mathematical rigor of numerical inverse design solvers while eliminating their expertise barriers through natural language comprehension and inference.

Nowadays, coding capabilities, including code generation, completion, analysis, and validation, have become one of the most crucial metrics of LLMs. While current state-of-the-art LLMs are already able to complete complex coding tasks, they face significant challenges when working with specialized solvers due to lack of domain-specific knowledge. Direct approaches to address this knowledge gap prove infeasible for two reasons: (1) many specialized solvers, particularly those in active development, are too recent or niche to be included in LLMs training data; and (2) even when documentation exists, providing complete solver documentation alongside user queries would exceed practical token limitations and dilute the LLM’s attention, leading to degraded performance even within context windows. These constraints necessitate a dynamic information retrieval mechanism that allows LLMs to request specific information as needed. Traditional function calling approaches in LLMs suffer from fundamental architectural limitations that require platform-specific implementations with separate development efforts needed for different LLM providers [29], [30], leading to a significant development overhead on reinventing wheels and limiting the scalability across different artificial intelligence (AI) applications [31]. In contrast, the recently proposed Model Context Protocol (MCP), introduced by Anthropic in Nov. 2024, addresses these limitations through a standard client-server architecture utilizing JSON-RPC 2.0 protocols, where client applications (such as Claude Desktop APP and ChatGPT) communicate with specialized MCP servers [32], [33], [34]. The protocol defines several core primitives, including tools (executable functions or scripts), resources (context information), and prompts (reusable instructions). The standardized tool discovery mechanism of the MCP enables dynamic querying of available servers through uniform protocol layers, addressing the issues of the “M × N problem” that turns the needs of developing MxN integrations between M AI applications and N tools into an M + N combination [35]. This standardization is particularly valuable for scientific computing tasks, offering modularized diagram for researchers to develop domain-specific MCP servers that can work across different LLM applications with reproducibility. MCP’s adoption by major technology companies including Microsoft, Google, and Cloudflare demonstrates its emergence as a *de facto* standard of LLM-tool integration [36], [37].

In this work, we propose an MCP assisted LLM framework for the inverse design of metasurfaces with customized MCP servers for contextual resources and the AD based numerical solver, TorchRDIT [8], for flexible end-to-end design tasks. For readers unfamiliar with TorchRDIT, Supplementary Information S5 summarizes the formulation and the generic optimization loop; full derivations appear in Ref. [8]. Architecturally, our proposed MCP-LLM structure diverges from the retrieval-augmented generation (RAG) [38]. General RAG enriches LLM context through a vector similarity search over text embeddings, which the performance of the embedding confines the quality of LLM’s responses. The MCP, on the other hand, enables the LLM to actively dominate the tool-use paradigm through well-defined protocols, following the model’s comprehension and reasoning. This enables capabilities beyond single-directional information retrieval, including constraint validation, long-term memory, and iterative refinement guided by external numerical simulation tools. RAG can be viewed as an open-loop workflow: the model consults static documentation and examples and then synthesizes a program in a largely single-pass manner. In contrast, our MCP-based agent operates in a multi-closed-loop regime in which the model drafts code, obtains structured validator feedback from standardized patterns and checks, and iteratively repairs its program. The experiments below compare these orchestration modes under matched prompts. To the best of our knowledge, no systematic evaluation exists to date for MCP-enabled LLM orchestration of end-to-end inverse design workflows using automatic differentiable solvers. Our key insight is that LLMs, when equipped with appropriate tool access via MCP, can effectively manage the entire design pipeline by synthesizing documentation with established optimization patterns.

This study is organized as follows: Section 2 presents the architecture and methodologies of our MCP-enabled LLM framework, detailing the design principles, MCP server implementations, and prompt strategies. We describe how the framework leverages template and documentation servers to provide autonomous access to TorchRDIT resources, enabling natural language orchestration of inverse design workflows. Section 3 provides comprehensive experimental results and analysis based on 100 trials of Huygens meta-atom inverse design tasks. We examine overall performance metrics, workflow efficiency, design quality, and error patterns to evaluate the effectiveness of different prompting strategies. Section 3 also reports a documentation-only RAG baseline under a matched structured prompt and a cross-orchestration comparison with the MCP framework. Finally, Section 4 concludes with a discussion of the framework’s implications for democratizing access to advanced computational design tools.

## 2 Methods

### 2.1 LLM-MCP design framework

Our framework prioritizes simplicity and accessibility over complexity. Instead of developing multi-agent systems or specialized LLM architectures, we created a minimalist approach where any MCP-compatible LLM can autonomously orchestrate inverse design workflows. Our technical contribution focuses on the MCP server infrastructure – specifically template and validation application programming interfaces (APIs) – making this an out-of-the-box template that researchers can adapt for their own solvers by simply creating domain-specific resources.

**Figure 1:**
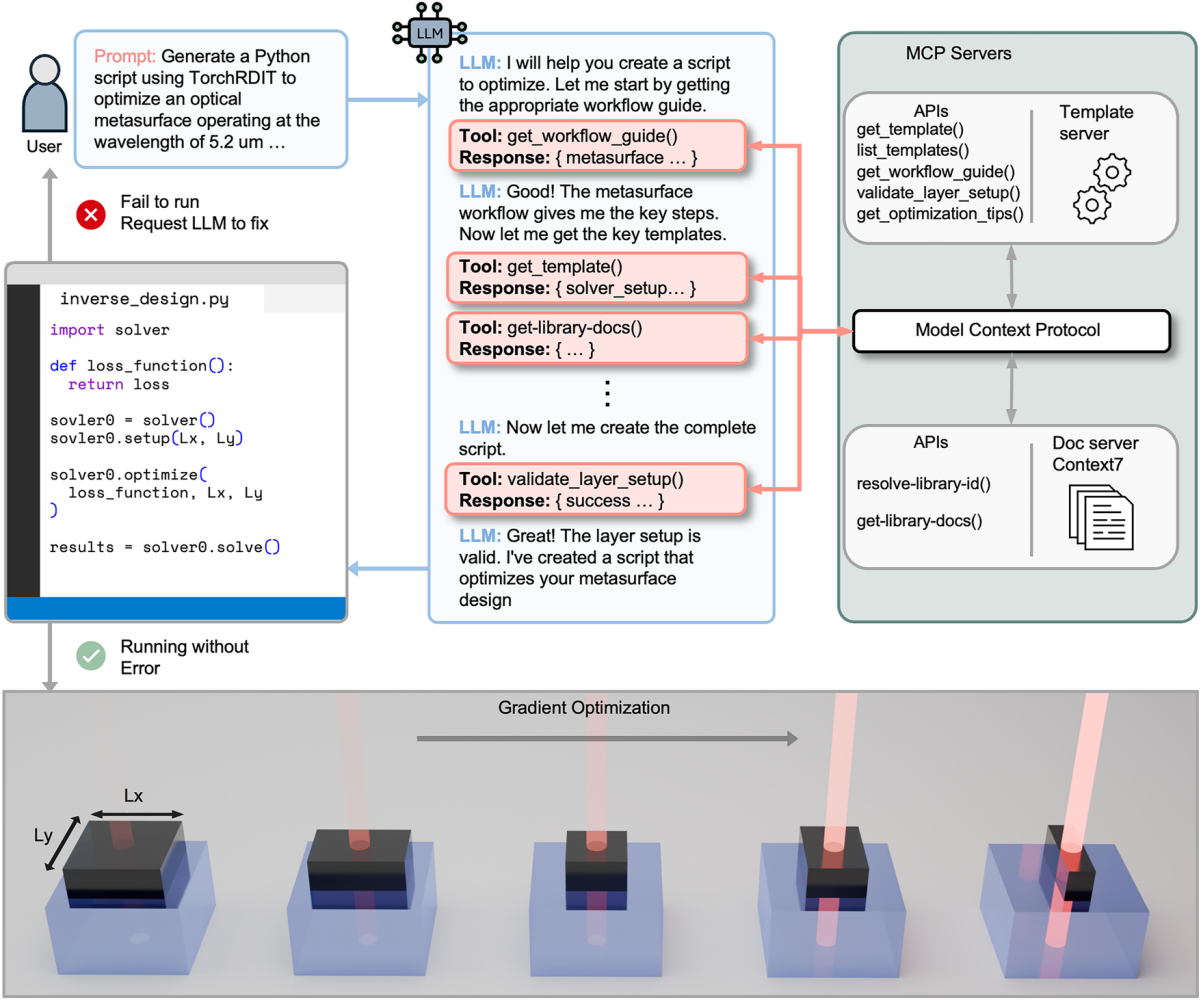
The schematic of the proposed LLM-MCP framework. Users provide queries that relate to the context of the problem and its design goals. The LLM analyzes queries with autonomous access to the provided MCP resources until enough information is obtained. The LLM generates executable Python code that implements the complete inverse design pipeline. A feedback loop enables interactive refinement based on execution results.

Figure 1 illustrates the framework’s operation. The LLM, acting as an MCP client, autonomously accesses well-structured APIs through MCP based on the JSON-RPC 2.0 protocol [32]. Without prescribed rules or hard-coded logic, the LLM decides whether to search documentation, retrieve templates, or combine both approaches based on its reasoning. This flexibility enables adaptation to varying problem complexities while maximizing model capabilities. Users can iteratively refine results through a feedback loop, improving generated solutions within the design session.

### 2.2 Documentation and templates for MCP servers

The LLM’s ability to generate accurate code for inverse design tasks depends critically on access to both working examples and accurate API documentation. We implement this through two complementary MCP servers: a documentation server that delivers up-to-date API references, and a custom template server that provides code patterns for TorchRDIT. The MCP server acts as an adaptor. It does not change solver capability. Tasks that exceed rigorous coupled-wave analysis (RCWA) or rigorous diffraction interface theory (R-DIT) scope would use additional algorithms or another solver exposed as a new resource.

To equip the LLM with comprehensive documentation resources and understand the available functions and parameters, an MCP server offering searching capabilities to the LLM is needed. Rather than developing a dedicated documentation server, we utilize Context7 [39], an existing documentation service that provides search capabilities for software libraries, as our documentation server. To optimize documentation retrieval, we structured the documentation of TorchRDIT according to search-friendly principles. Each documentation page includes structured metadata (title, category, tags, complexity level), consistent section organization, and dedicated search keywords. In addition, code snippets for all functions and features are provided, along with examples. These principles ensure that whichever piece of information the LLM fetches, there will be sufficient context surrounding it, reducing the model’s hallucinations.

While this framework requires no specific number of MCP servers or APIs to function, our proposed customized template server with 5 APIs addresses a fundamental limitation: LLMs with documentation access alone often fail to grasp domain-specific patterns, leading to conceptual errors and misunderstanding. For example, in our specific cases, LLM may only search for basic usages and implement manual finite differences for optimization instead of using built-in automatic differentiation. Table 1a summarizes the 5 core APIs exposed to the LLM. We develop all APIs using the tools primitive since it is the most supported feature across the existing clients in the community. These APIs are designed to prioritize self-contained executability, extensive inline documentation, and proactive error prevention. Our template server follows several design principles: (1) Modular workflow exposure: list_templates provides categorized examples without overwhelming token limits; (2) On-demand retrieval: LLMs fetch only relevant patterns for specific task; (3) Proactive validation: validate_layer_setup catch common errors identified through pre-collected testing trials. The get_template API provides 16 verified code patterns covering common TorchRDIT coding examples, as shown in Table 1b. Each template contains working code with instructive comments that explain the code logic and the corresponding design parameters. The complete template snippets are demonstrated in Supplementary Information S4. The validate_layer_setup API proactively checks the generated code snippets for common mistakes such as incorrect layer stack order or wrong function calls, providing immediate feedback for corrections during the generation process. This way of designing APIs stems from observing LLM behaviors. For generalizing to other simulation tools, we recommend starting with documentation-only access, identifying systematic LLMs failures through trials and testing, and then creating a dedicated APIs of the MCP servers that embed domain expertise.

**Table 1:** TorchRDIT template MCP API overview.

(a) Summary of TorchRDIT template MCP API endpoints		
API endpoint	Description	Parameters
get_template	Retrieves code template	template_name (str)
list_templates	Lists available templates	category (str, optional)
get_workflow_guide	Provides workflow instructions	workflow_type (str)
validate_layer_setup	Validates code for errors	code_snippet (str)
get_optimization_tips	Returns optimization guidance	None

(b) TorchRDIT MCP template categories and examples			
Category	Template count	Examples	Focus area
Basic	11 templates	solver_setup, layer_stack	Core TorchRDIT functionality
Optimization	2 templates	gradient_based, multi_objective	Inverse design patterns
Clarifications	3 templates	layer_order, common_mistakes	API corrections and pitfalls

Notably, while our optimization templates (in Table 1b) leverage TorchRDIT’s differentiable feature, which is the unique functionality of this solver and may not be applicable in other solvers, the framework architecture itself remains solver-agnostic with the consistent modular template discovery, selective retrieval, and validation. Other non-differentiable solvers would expose different optimization strategies through their templates with best practices.

These two MCP servers operate synergistically without prescribed coordination rules. The LLM autonomously decides whether to search documentation, retrieve templates, or combine both approaches based on its ongoing reasoning states. With accessible information, the quality of the generated codes is therefore determined by the performance and tool usage of the language model.

### 2.3 Prompt strategies and evaluation task

To evaluate the effectiveness of our proposed framework, it is applied to address a challenging optical Huygens meta-atom inverse design task using two different prompt strategies [8]. A concise summary of the metasurface platform and parameter conventions is provided in Supplementary Information S6. This task verifies whether LLMs can autonomously complete the coding for the inverse design optimization when given the MCP resources, and how explicit prompt guidance affects the quality and reliability of generated solutions.

First, we use a natural language prompt (P1) to describe the design task and the design requirements: “Generate a Python script using TorchRDIT to optimize an optical metasurface operating at the wavelength of 5.2 um. The metasurface consists of a grating layer on top and a substrate. The grating layer (650 nm) is a two-layer PbTe model (top half layer: n_top = 4.8; bottom half layer: n_bottom = 5.4, k_bottom = 0.01). The substrate is CaF2 (n_caf2 = 1.4). The periodicity is 2.5 um in both the *x* and *y* directions. The incident light (TM mode, *x*-polarized) is transmitted from the substrate and out of the top grating layer to the air in the normal direction. The grating layer is a rectangular pillar, and its length and width are to be optimized by TorchRDIT to achieve a transmission efficiency greater than 80 % while also meeting a target transmitted TM phase of 170°. The relative errors of phase in degrees should be less than 5 %. Use Context7 to search the docs of TorchRDIT and use torchrdit-mcp to get coding templates.” The LLM receives only the design requirements without workflow instructions or optimization strategies. Because P1 specifies only the design goals and no workflow, the LLM is free to choose different tool sequences and API calls across trials, which leads to variability in the generated code and outcomes even for identical prompts (see Table S2). We use this prompt to test whether the model can autonomously complete the coding for the inverse design optimization given only the design requirements.

In contrast to the minimalist natural language prompt, we also provide a more explicit prompt (P2) that features advanced prompt engineering techniques (see Supplementary Information S1 for the full prompt). This prompt begins with role prompting, establishing a “TorchRDIT Design Assistant” persona with domain expertise. A workflow prompt implements task decomposition through a prescribed 7-step process, effectively providing chain-of-thought scaffolding that guides the LLM through complex reasoning steps from goal clarification to code delivery [40]. Central to P2 are instructive optimization strategies, which include a mandatory two-stage global search that combines parameter sweep with gradient refinement. It first performs a parameter sweep to obtain good initial values, then runs gradient refinement. This reduces sensitivity to initialization and lowers the variance of results. This part of the prompt employs both algorithmic and tool-augmented prompts, explicitly instructing when to call specific tools and what computational strategies to use. Finally, quality assurance is also ensured through code implementation constraints (including instructions on code structure, error handling, and output format) and a validation checklist, which provides a defensive programming pattern.

By comparing P1 and P2, two fundamental questions can be answered: can LLMs independently discover and implement best practices for inverse design optimization? Does explicit guidance significantly improve the quality of outcomes? To disentangle prompting effects from orchestration, we also define P2-R, a structured prompt used with the documentation-only RAG baseline. P2-R mirrors the workflow wording of P2 while learning TorchRDIT API usage from retrieved sources at run time rather than from executable templates. The full wording of P2-R is provided in Supplementary Information S7.2.

### 2.4 Baseline comparison with RAG

We construct a documentation-only retrieval baseline to isolate orchestration effects. The same model and sampling settings as in the MCP experiments are used. The corpus comprises TorchRDIT documentation pages at the method level, including examples, and excludes the template modules designed for the MCP server. The prompt follows P2-R, which aligns its workflow wording with P2 while learning actual API names and signatures from retrieved sources during the run. Platform choices, corpus composition, retrieval and ranking settings are specified in Supplementary Information S7. The evaluation setup and metrics are described in Section 3.

## 3 Results

### 3.1 Experimental overview and metrics definition

In this work, we conducted 50 trials for each prompt strategy, namely P1, P2, and with P2-R for RAG comparison, using Claude Sonnet 4 on Claude Desktop APP to assess the effectiveness of our proposed inverse design optimization, with TorchRDIT 0.1.20 for running the actual inverse design tasks. We statistically examine overall performance, workflow efficiency, design quality, and failure patterns to systematically characterize the framework’s functionality and LLM-MCP interaction dynamics. All statistical comparisons between prompt strategies employ the Mann-Whitney U test for continuous variables and the chi-square test for categorical distributions, with Cohen’s *d* calculated to quantify effect sizes.

To gain a better view of the design quality regarding both optimization objectives (transmission efficiency and phase), we define a composite scoring metric that combines transmission efficiency and phase accuracy into a single normalized score. The composite score *S* ∈ [0, 1] is calculated as the weighted average of individual objective scores:

(1) $$S=w_{T}\cdot S_{T}+w_{\phi }\cdot S_{\phi },$$

where *S*_*T*_ is the transmission score, *S*_*ϕ*_ is the phase score, and *w*_*T*_ = *w*_*ϕ*_ = 0.5 represents equal weighting between objectives. Higher composite scores indicate better design quality. The transmission score *S*_*T*_ uses a piecewise function that assigns 0.5 at the minimum requirement threshold of 80 % and scales linearly above and below:

(2) $$S_{T}=\left\{\begin{array}{cc} 0\; & \;\text{if }T<0\text{ or missing},\\ 0.5\cdot \frac{T}{0.8}\; & \;\text{if }0\leq T<0.8,\\ 0.5+0.5\cdot \min\left(1,\frac{T-0.8}{0.2}\right)\; & \;\text{if }T\geq 0.8, \end{array}\right.$$

where *T* is the transmission efficiency. This definition ensures designs meeting the minimum requirement (*T* ≥ 0.8) receive a score of 0.5, with linear scaling to 1.0 at 100 % transmission. The phase score *S*_*ϕ*_ penalizes deviation from the target phase of 170° with stricter scoring that emphasizes precision:

(3) $$S_{\phi }=\left\{\begin{array}{cc} 0\; & \;\text{if missing},\\ 1-0.5\cdot \frac{\left|\phi_{\text{error}}\right|}{8.5{}^{\circ}}\; & \;\text{if }|\phi_{\text{error}}|\leq 8.5{}^{\circ},\\ 0.5\cdot \exp\left(-\frac{|\phi_{\text{error}}|-8.5{}^{\circ}}{10{}^{\circ}}\right)\; & \;\text{if }|\phi_{\text{error}}|>8.5{}^{\circ}, \end{array}\right.$$

where *ϕ*_error_ = |170° − *ϕ*_actual_| is the phase difference between the target phase and the actual phase. Designs within tolerance (±8.5°) receive a score from 0.5 to 1.0, while those outside tolerance decay exponentially towards 0. The classification of design quality levels are demonstrated in Table S1 in the Supplementary Information.

Based on the composite score, we further define design efficiency score (DES) as the ratio of the composite score to the number of conversations turns to evaluate the efficiency:

(4) DES=composite score/number of conversation turns.

To characterize error feedback dynamics across interaction steps, we report a turn-level error profile *P*_*t*_ defined as below:

(5) $$P_{t}=\frac{\#\;of\;trials\;that\;reached\;t\;and\;exhibited\;an\;\left[error\;type\right]\;at\;t}{\#\;of\;trials\;that\;reached\;t},$$

where *t* ∈ {0, …, 5}, and the error type is defined in Table S4. The denominator in (5) avoids bias from sessions that conclude earlier.

### 3.2 Workflow efficiency and tool usage patterns

The fundamental question driving our investigation is whether LLMs can successfully generate working codes that can run complete inverse design optimization without any errors. We do not expect the code generation to be completed by a single query, and some errors are likely to occur in the generated code. In our analysis, we allow the user at most five attempts (5 conversation turns) to request corrections from the LLM to correct the errors. When the LLM cannot fix all errors in the five following queries, or the generated codes deviate from the design requirements that are hard to fix with only simple instructions, we consider the trial as a failure.

**Figure 2:**
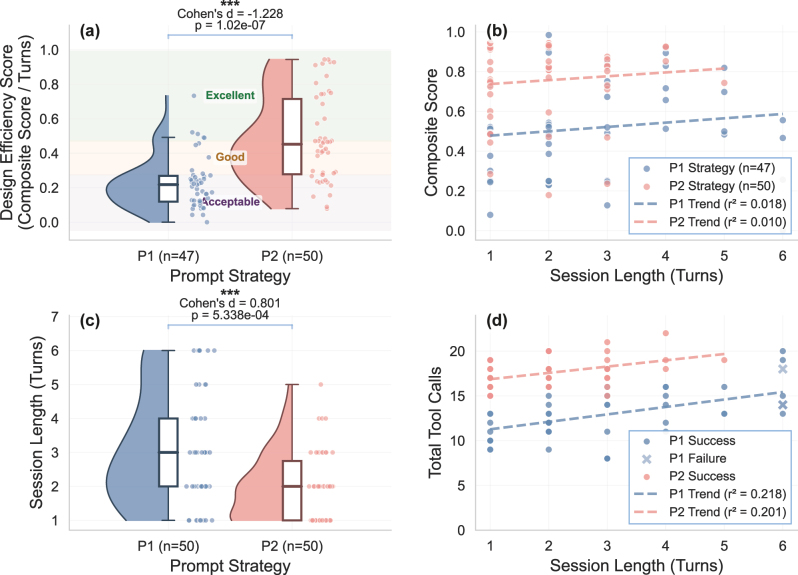
Workflow efficiency metrics and design efficiency score (DES) analysis. (a) DES distributions with quartile-based performance zones. Excellent: top 25 % of DES values; good zone: 50th - 75th percentile; acceptable: bottom 50 % of DES values. (b) Composite score versus session length. (c) Session length distributions. (d) Tool usage analysis versus session length.

Figure 2a statistically reveals highly significant differences of DES between the two prompt strategies (*p* < 0.001, |*d*| = 1.228), with P2 achieving higher mean efficiency than P1 (0.48 vs. 0.23). From the total 100 experimental trials, there are 47 successful trials for P1 and 50 successful trials for P2. The basic experimental statistics are shown in Table S2. Both strategies show a high success rate within the 5-attempt limit. The design quality of P2 is significantly higher than that of P1. The P2 strategy achieves a 76 % satisfaction rate in meeting both transmission and phase requirements, while P1 only achieves 23 %. The 3 failing cases are marked because generated codes are not fixed by the LLM within the max attempts limits, whose failure reasons include: vanishing gradient problem due to setup issues and new runtime errors introduced during fixing attempts.

The quartile-based performance zones shown in Figure 2a show that P1 clusters below median performance while P2 extends into the excellent zone, indicating superior quality per turn achievement. The regression analysis in Figure 2b illustrates different convergence behaviors. P1’s weak positive correlation (*r* ^2^ = 0.018) suggests that quality improvement requires extended iteration, whereas P2’s consistent high-performance clustering demonstrates rapid convergence to optimal solutions, typically within the first three iterations.

Session length distributions in Figure 2c demonstrate good predictability of P2. The concentrated distribution of P2 over shorter session lengths suggests that structured prompt techniques can significantly reduce the number of turns required to achieve a satisfactory design, in contrast to P1’s extended tail, which requires further user queries. Figure 2d shows weak correlations between tool usage patterns and session length for both prompt strategies. P2’s lower correlation coefficient (*r* ^2^ = 0.201) compared to P1 (*r* ^2^ = 0.218) suggests that P2 has more efficient tool utilization, achieving higher outcomes with more focused engagement rather than extensive exploration of tool usage, as seen in P1.

**Figure 3:**
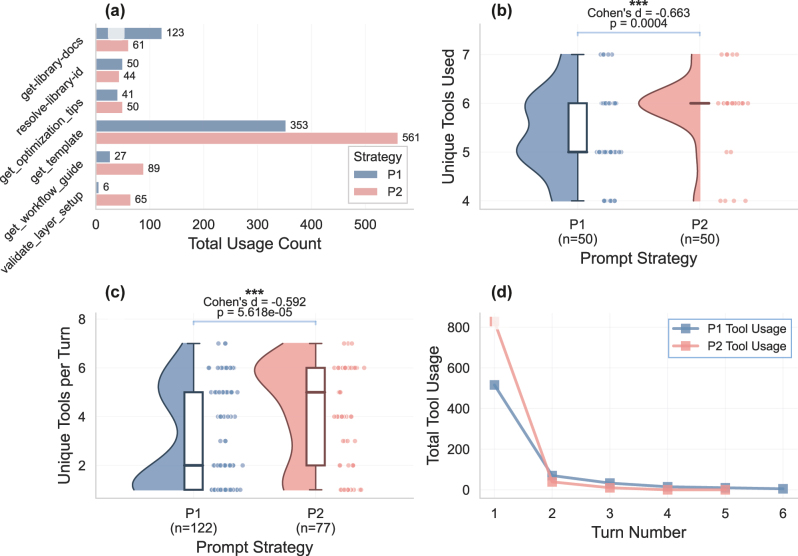
Comprehensive tool usage pattern analysis across prompt strategies. (a) Top 6 tool usage analysis. (b) Tool diversity per trial. (c) Average tools per turn analysis. (d) Temporal tool usage patterns aggregated by turn number.

In addition to analyzing work efficiencies, understanding tool utilization patterns is also important to understand how prompt strategies affect the model’s decision-making process. Figure 3a establishes a clear preference structure across strategies. The most frequently used tool, get_template, is utilized 914 times in total, with P2, which includes structured guidance, having a higher usage rate (561 uses) compared to P1, which has 353 uses. This pattern indicates that the model under the prompt P2 tends to acquire more information from templates. Other tools, such as get-library-docs, show more usage in P1, indicating a more passive approach to data acquisition, including searching during the first turn generation and the issue-solving turns. Notably, the usage of validate_layer_setup shows extreme strategic divergence with P2 using it 65 times compared to P1’s 6 times. This suggests that P2’s model is more proactive for conducting validation-based error checking following the prompt, while P1 relies on model’s self-correction even with the exposed tool information.

Tool diversity analysis (Figure 3b and c) reveals that the model under P2 employs diverse tool access patterns, with a tighter distribution around 6 unique tools per trial and a median of 5 tool calls per turn. The violin plots indicate that the structured prompt strategy with explicit guidance yields a more consistent and stable tool usage pattern, resulting in a more comprehensive information gathering process and validation testing at each workflow decision point.

Temporal dynamics, as shown in Figure 3d, reveal a dramatic front-loaded exploration with peak usage at turn 1 (P1: 520, P2: 810 tools) followed by rapid exponential decay to near-zero by turn 4. This rapid decay suggests that most critical information gathering and setup occurs in the initial turns, with later turns focused on refinement rather than exploration. It also shows that the model under P2 tends to call more tools in the early stages of the design process, whereas P1’s model requires more iterations with additional tool calling to revisit APIs that were not correctly used in previous turns.

**Figure 4:**
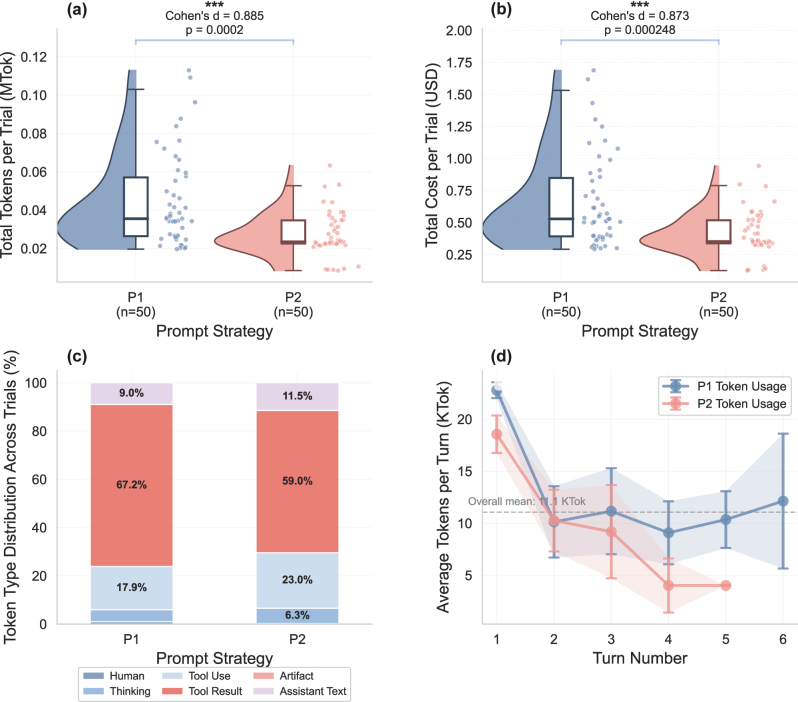
Token usage and cost analysis across prompting strategies. (a) Distribution of estimated total tokens consumed per trial showing P2’s more efficient token usage (Cohen’s *d* = 0.885, *p* < 0.001). (b) Total computational cost per trial in USD (input token: $3/MTok, output token: $15/MTok), with P2 achieving 37 % cost reduction. (c) Token type distribution across all trials, revealing similar proportions of tool usage (P1: 17.9 %, P2: 23.0 %) and tool results (P1: 67.2 %, P2: 59.0 %). (d) Temporal dynamics of token consumption per turn, showing front-loaded usage patterns with P2 maintaining lower consumption throughout the conversation.

We further analyzed token usage patterns and associated costs across both prompting strategies (Figure 4). The token analysis reveals that P2’s structured guidance not only improves design quality but also reduces computational overhead. Panel (a) shows the distribution of total tokens consumed per trial. P2 demonstrates lower token usage (0.03 ± 0.01 million tokens) compared to P1 (0.04 ± 0.02 million tokens). This 25 % reduction in token consumption directly translates to cost savings, as shown in Figure 4b, where P2 trials cost an average of $0.41 ± 0.17 compared to P1’s $0.66 ± 0.36 – a 37 % reduction in computational expense.

The token type distribution (Figure 4c) provides insights into how the LLM allocates its computational resources. Both strategies show similar patterns, with tool results comprising the majority of tokens (P1: 67.2 %, P2: 59.0 %), followed by tool use calls and assistant-generated text. Notably, P2 shows a higher proportion of tool use tokens (23.0 % vs. 17.9 %), suggesting more efficient and targeted tool utilization rather than excessive result processing.

The temporal dynamics of token consumption, as demonstrated in Figure 4d, reveal distinct patterns between strategies. Both exhibit front-loaded token usage, with the highest consumption in the first turn (P1: 23K tokens, P2: 19K tokens) as the LLM explores available tools and establishes the design approach. However, P2 maintains consistently lower token usage across all turns and shows a steeper decline, stabilizing at approximately 4K tokens per turn by turn 4, while P1 reaches around 10K tokens. This pattern aligns with our earlier findings that P2 requires fewer conversation turns overall and fewer document and template information requests for fixing coding issues, suggesting that structured guidance enables more efficient problem-solving trajectories.

These patterns demonstrate that structured prompting (P2) simultaneously improves design quality while reducing computational costs and user interaction time. The front-loaded tool usage indicates that explicit structure enables LLMs to gather necessary information upfront rather than through iterative exploration, resulting in fewer conversation turns, lower token consumption, and reduced latency – critical advantages for practical deployment in resource-constrained applications.

### 3.3 Performance and design quality

**Figure 5:**
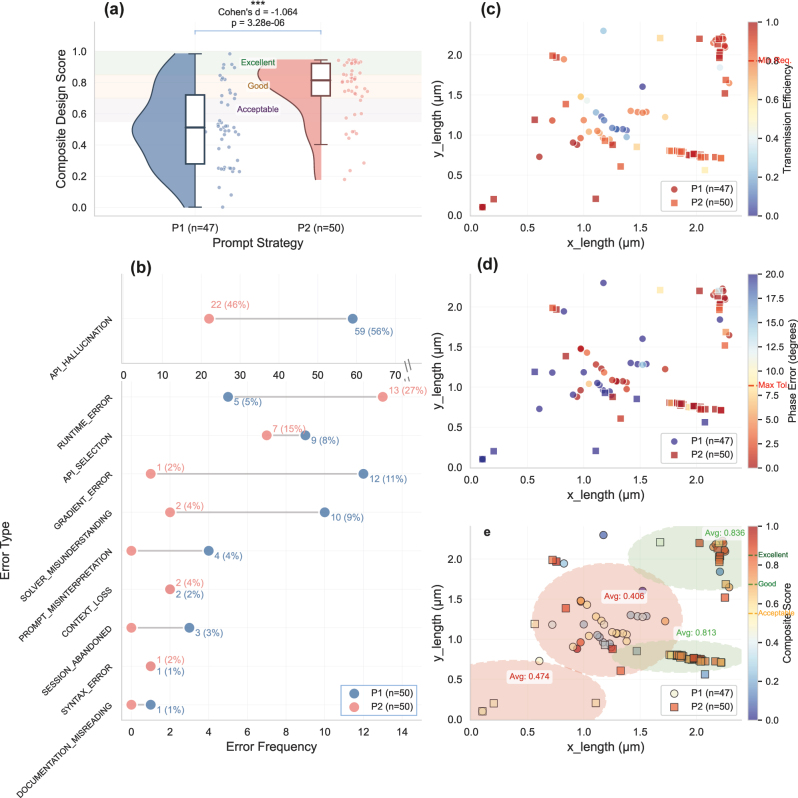
Performance and design metrics between natural language (P1) and structured guidance (P2) prompt strategies. (a) Composite score distribution of all successful trials versus prompt strategies. The composite score classification is shown in Table S1 in Supplementary Information. (b) Absolute counts shown with percentages of total errors (P1 = 106, P2 = 48). Scatter plots show (c) transmission efficiency, (d) phase error, and (e) composite score as functions of x_length and y_length for prompting strategies P1 (circles, *n* = 47) and P2 (squares, *n* = 50). Red dashed lines indicate design requirements. Panel (e) includes performance-based clustering with average scores annotated for each region.

Beyond the previous investigation of the effectiveness, understanding the quality of outcomes delivered by the LLM under different prompt strategies also provides crucial insights. The composite score distribution, as shown in Figure 5a, illustrates that P2 designs show a clear shift toward higher scores with a median of 0.756 compared to only 0.488 for P1. The combination of very small *p*-values (<1e-5) with large effect sizes (Cohen’s *d*: |*d*| > 0.8) provides strong evidence that P2 strategy produces meaningfully better design outcomes, not just statistically detectable differences.

The spatial distribution of performance metrics across the optimized width and length (x_length and y_length in Figure 5c–e) reveals distinct optimization patterns. Without detailed optimization guidance from P2, the LLM tends to code with basic gradient optimization flow, which relies heavily on the initial values, and is easy to be trapped in the local minimum, such as the low transmission efficiencies cluster (blue circles at the center) in Figure 5c. With pre-searched candidates of the high-performance initial points under P2, the outcomes show more consistent and focused in the global minimum as shown in the two clusters with green zones in Figure 5e. Table S3 in Supplementary Information show 6 designs with top performance grouped by prompt strategies. Examples of the generated scripts by two prompt strategies (P1-R06 and P2-R40) can be found in Supplementary Information S3.

### 3.4 Error analysis

Having demonstrated the performance and efficiency analysis, we now examine the specific error patterns that emerged during the trials to understand the model’s behavior and the impact of the prompt strategies. To systematically analyze failure modes during the trials, we identify and categorize ten distinct error types encountered when running the generated codes. Table S4 in Supplementary Information summarizes the definitions of these error types.

Figure 5b illustrates the frequency distribution of these error types across both prompting strategies. Among all trials with both strategies, API_HALLUCINATION emerged as the dominant error type. With the structured guidance (P2), the absolute count of API_HALLUCINATION is reduced to 1/3 of that with P1, suggesting that the structured guidance (P2) provides more accurate information and a more reliable reasoning pattern for the LLM to generate the correct code. It is also worth noting that the error type of API_SELECTION contains both APIs of TorchRDIT and other libraries, and P2 reduces it through two primary mechanisms: (1) it prevents API hallucination by providing explicit examples of TorchRDIT, reducing the LLM’s tendency to generate plausible but non-existent APIs; (2) it maintains conceptual consistency through the task by enforcing a logical progression and task decomposition so that both APIs of TorchRDIT and other libraries are well constrained.

Conversely, P2 shows a higher count of RUNTIME_ERROR, which is due to the more complex optimization coding patterns with P2 compared to the simpler coding patterns with P1. Most notably, P2 nearly eliminates errors of SOLVER_MISUNDERSTANDING, PROMPT_MISINTERPRETATION, and GRADIENT_ERROR. The recovery of these errors requires users with a clear understanding of either the TorchRDIT’s APIs or the gradient-based optimization principles, indicating the effectiveness of the structured guidance (P2) in reducing users’ cognitive load when using the proposed framework.

These findings validate the importance of structured prompting in the TorchRDIT framework design. By providing scaffolding that aligns with the solver’s workflow – from geometry definition through material assignment, source configuration, and optimization – the framework guides users toward successful implementations while preventing the most problematic error types. This error analysis thus provides empirical justification for the framework’s emphasis on structured, template-based interactions over free-form natural language queries.

### 3.5 Baseline analysis between MCP and RAG

Using the same model and the evaluation process specified in the previous sections, the documentation-only retrieval baseline with a structured prompt (RAG + P2-R) achieved 1 of 50 successful trials. Success rate and the DES value for that single success are summarized together with MCP + P2 in Table 2.

**Table 2:** DES metrics and success rates by strategy.

Strategy	Trials		Success rate	DES			
	Total	Successful	(%)	Mean	Median	Std	Samples
MCP + P2	50	50	100.0 %	0.48419	0.45269	0.24827	50
RAG + P2-R	50	1	2.0 %	0.15338	0.15338	0.00000	1

**Figure 6:**
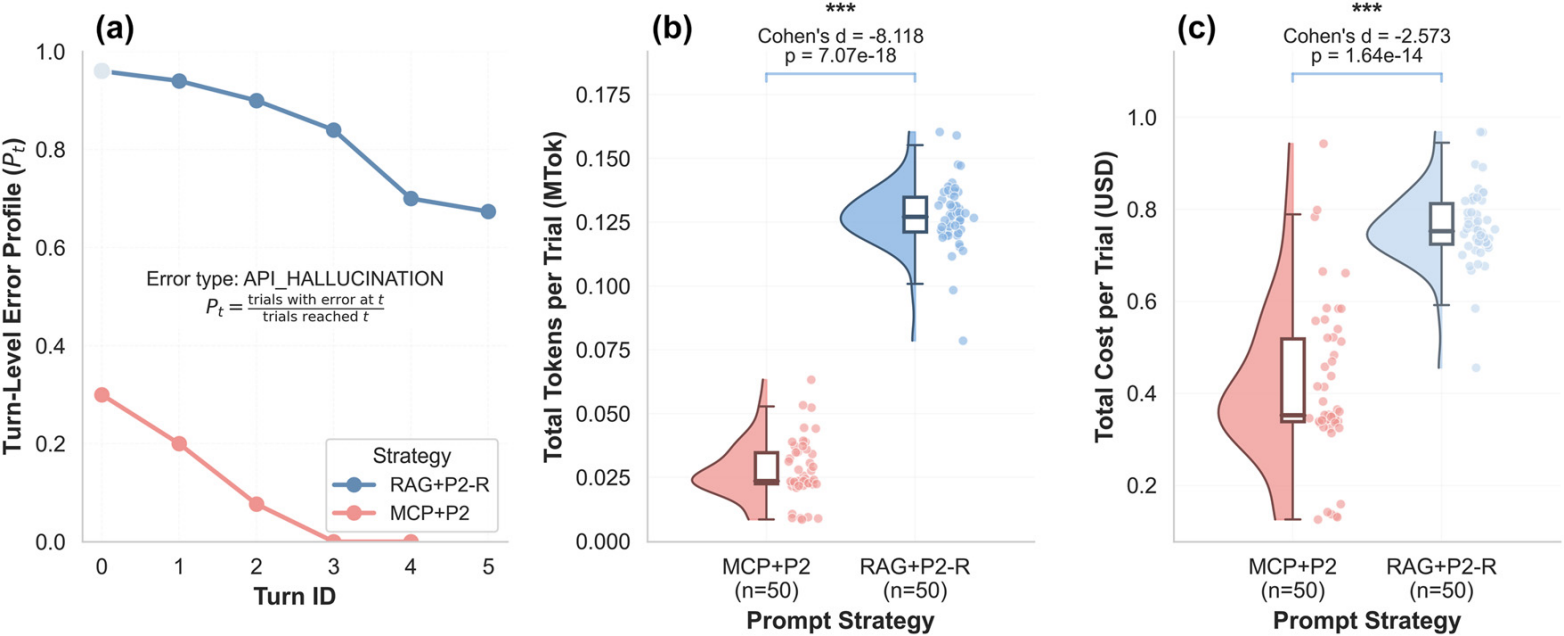
Cross-orchestration benchmarks under matched structured prompts. (a) Turn-level error profile for API_HALLUCINATION. Each point shows the fraction of trials that reached a given turn and exhibited the error event on that turn. (b) Total tokens per trial (MTok) with summary statistics and Cohen’s *d* annotated above the comparison. (c) Total cost per trial (USD) computed using the same token pricing as Figure 4, with summary statistics and effect size annotated above the comparison.

As shown in Table 3 and Figure 5b, API_HALLUCINATION is the largest category for both methods, which motivates the focus on a further analysis in Figure 6a.

**Table 3:** Distribution of error types in RAG + P2-R turns.

Error type	P2-R turns		Error type	P2-R turns	
	Count	%		Count	%
API_HALLUCINATION	250	83.6 %	RUNTIME_ERROR	21	7.1 %
API_SELECTION	14	4.7 %	GRADIENT_ERROR	7	2.3 %
SESSION_ABANDONED	3	1.0 %	SOLVER_MISUNDERSTANDING	1	0.3 %
CONTEXT_LOSS	1	0.3 %	SYNTAX_ERROR	1	0.3 %

Figure 6a presents the turn-level error profile *P*_*t*_ for API_HALLUCINATION. Errors appear in the first and second turns for both methods. Under MCP + P2, the profile drops to near zero in subsequent turns, consistent with a retrieve, assemble, validate, and repair loop that is augmented by offline execution feedback supplied by the user between turns. Under RAG + P2-R, the profile remains high through turn 5, which indicates persistent failures in the absence of executable validation.

Figure 6b and c compare total tokens and total cost per trial, computed as in Figure 4. RAG + P2-R consumes more tokens and cost per trial while rarely reaching a valid evaluation. Cohen’s d and the corresponding p values are annotated above each comparison. Representative chat logs for a convergent MCP + P2 session and a non-convergent RAG + P2-R session are provided in Supplementary Information S8.

Conceptually, the documentation-only RAG agent follows an open-loop, single-pass pipeline of knowledge retrieval and inference. Its retrieval configuration and reasoning plan are fixed at design time, so later turns mainly revise prior code with limited new information. In contrast, MCP enables an LLM oriented workflow in which the model selects at run time which documentation to consult, which verified templates to assemble, and when to request static validation through validate_layer_setup. Within a single session, the model performs repeated cycles of retrieve, assemble, validate, and repair, and structured feedback arises from validator messages. This difference aligns with the fast drop of the turn level profile for MCP + P2 in Figure 6a and with the lower interaction cost in Figure 6b and c.

## 4 Conclusion and discussion

This work demonstrates that LLMs equipped with MCP tool access can effectively orchestrate complex inverse design workflows while maintaining mathematical rigor. Both prompting strategies achieved high success rates (P1: 94 %, P2: 100 %), validating autonomous code generation capability. A further comprehensive comparison shows that structured guidance (P2) significantly outperformed natural language prompts (P1) with a 3.3× improvement in satisfaction rate (76 % vs. 23 %) and 2.1× improvement in DES (0.48 vs. 0.23). Moreover, P2 achieved these quality improvements while reducing computational costs by 37 % through more efficient token usage, demonstrating that better prompting strategies can simultaneously enhance performance and efficiency. Error analysis revealed that P2 reduced API hallucinations by 67 % and nearly eliminated solver misunderstanding errors – the primary barriers for non-expert users. By combining numerical solver rigor with natural language understanding through standardized MCP, our framework enables researchers to focus on scientific innovation rather than implementation details, establishing a practical paradigm for democratizing access to specialized computational tools. Under a matched structured prompt, the documentation-only RAG baseline shows markedly lower reliability and efficiency. In our setting, the MCP agent assembles verified templates and requests static validation through validate_layer_setup, which provides actionable feedback for iterative repair without executing the program in the session; the cross-orchestration comparison in Figure 6 and Table 3 reflects this difference.

Our minimalist approach – 5 core APIs without complex multi-agent systems – provides an adaptable template for computational tools beyond TorchRDIT. The design principles (modular workflow exposure, on-demand retrieval, proactive validation) guide integration of other specialized solvers with LLMs. While our templates use TorchRDIT’s differentiable features, the framework architecture remains solver-agnostic; non-differentiable solvers can expose their optimization strategies through similar structures. Within TorchRDIT, which implements a Fourier modal solver for periodic unit cells, scaling to multi-parameter metasurfaces is handled by the same workflow by enlarging the design vectors. For large-area, spatially varying devices such as metalenses, standard components such as local periodic approximation and free-space field propagation can be surfaced as additional templates or as MCP resources to an external solver, while the MCP layer and prompting strategy remain unchanged. The success of the framework demonstrates that effective LLM-assisted scientific computing requires a thoughtful interface design aligned with tool workflows. Although domain knowledge helps users formulate requirements and interpret results, the framework significantly reduces expertise barriers for AD-based design tools. Future advances in LLMs and agentic frameworks will enable more automated capabilities for complex tasks such as multiphysics simulations and coupled optimization problems.

## Supplementary Material

Supplementary Material Details
