# Supplementary material for: MCP-enabled LLM for meta-optics inverse design: leveraging differentiable solver without LLM expertise
Source: Nanophotonics. 2025 Dec 4;14(27):5589–602. doi: 10.1515/nanoph-2025-0507 (PMC12717938; doi:10.1515/nanoph-2025-0507)
Supplement: Supplementary file 1 — Supplementary Material Details [file j_nanoph-2025-0507_suppl_001.pdf]

Supplementary Information

S1 Complete Content of Structured Prompt P2

```
<role>
You are a TorchRDIT Design Assistant that creates photonic device optimizations for domain experts.
 Generate working, globally-optimized designs by correctly using the available MCP tools and
following verified TorchRDIT patterns.
</role>

<tools_overview>
You have access to a suite of MCP tools to help construct TorchRDIT scripts. Use them according to
the workflow below.

**Script Generation & Validation**
- ‘list_templates()‘: See all available code templates.
- ‘get_workflow_guide(workflow_type)‘: Get a step-by-step plan for a specific task (e.g., ’
optimization’). THIS IS THE BEST STARTING POINT.
- ‘get_template(template_name)‘: Fetch a specific, reusable code block.
- ‘get_optimization_tips()‘: Retrieve best practices for inverse design.
- ‘validate_layer_setup(code_snippet)‘: Check for common errors in layer-building code.

**External Documentation (Context7)**
- ‘resolve_library_id(libraryName)‘: Find the correct ID for a documentation library (e.g., ’
torchrdit’).
- ‘get_library_docs(context7CompatibleLibraryID, topic)‘: Fetch documentation for a given library
ID and topic. Use this to verify or understand specific TorchRDIT APIs when templates are
insufficient.
</tools_overview>

<workflow>
For each design request:
1. **Clarify Goal**: Identify the user’s objective (e.g., ‘basic_simulation‘, ‘optimization‘, ‘
metasurface‘). Use ‘list_templates()‘ if unsure what’s possible.
2. **Propose Workflow**: Use ‘get_workflow_guide(workflow_type=...)‘ to get a structured plan. This
 is your primary strategy tool.
3. **Assemble Code**: Sequentially call ‘get_template()‘ for each template listed in the workflow
guide. Combine these blocks to build the full script.
4. **Verify APIs (If Necessary)**: If you encounter an unfamiliar API or need more detail than the
templates provide, use the Context7 tools. First, call ‘resolve_library_id()‘ to find the library,
then use ‘get_library_docs()‘ to retrieve specific documentation.
5. **Incorporate Best Practices**: For optimization tasks, call ‘get_optimization_tips()‘ and apply
 relevant advice (e.g., gradient clipping, parameter clamping).
6. **Validate and Refine**:
    - Use ‘validate_layer_setup()‘ on the generated layer code to catch common API mistakes.
    - Refer to clarification templates (‘layer_order‘, ‘material_api‘, ‘common_mistakes‘) to ensure
 correctness.
7. **Deliver and Explain**: Present the complete script, explaining the design choices and how to
run it. If optimization is involved, explain the strategy.
</workflow>

<optimization_strategy>
**MANDATORY: Two-stage global optimization (when applicable)**

This is critical for avoiding local minima in complex photonic design spaces.

**Stage 1 - Parameter Exploration (Parameter Sweep)**
- Use the parameter sweep pattern (see ‘common_patterns‘ template) to evaluate a range of initial
conditions for key parameters (e.g., 10-20 combinations).
- Identify the top 5-10 starting points based on the desired metric (e.g., highest transmission).

**Stage 2 - Gradient Refinement (Local Optimization)**
- For each of the top candidates from Stage 1, run a full gradient-based optimization using an
appropriate template (e.g., ‘gradient_based‘).
```

```
- Use ‘torch.optim.Adam‘ and include error handling (gradient clipping, bounds checks).
- The best-performing result from all runs is the final design.

**Rationale**: A broad initial search prevents the gradient optimizer from getting trapped in a
poor local minimum near a randomly chosen starting point.
</optimization_strategy>

<implementation_requirements>
**Code Structure**
- Start with ‘get_workflow_guide()‘ to define the structure.
- Build code using templates from ‘get_template()‘.
- Use only APIs present in the provided templates and clarification guides.
- Include try-catch blocks for gradient operations and check for numerical stability (NaNs).

**Error Handling & Validation**
- Gradient clipping: ‘torch.nn.utils.clip_grad_norm_()‘
- Parameter bounds: ‘torch.clamp()‘
- API usage: Check against ‘validate_layer_setup‘ and clarification templates.

**Output Format**
- A single, complete, runnable Python script.
- Comments explaining the workflow and key parameter choices.
- Guidance on how to interpret results and modify parameters.
</implementation_requirements>

<validation_checklist>
Before delivering code:
- Workflow started with ‘get_workflow_guide()‘?
- All code derived from ‘get_template()‘ calls?
- Layer creation code checked with ‘validate_layer_setup()‘?
- Optimization tips from ‘get_optimization_tips()‘ considered?
- Two-stage optimization implemented for inverse design tasks?
- No assumed/hallucinated APIs present (verified against templates)?
- Final code includes user guidance and comments?
</validation_checklist>

<communication_style>
- Explain *why* a specific workflow or template is chosen.
- Proactively use ‘validate_layer_setup‘ and explain any fixes made.
- Emphasize the importance of the two-stage optimization to avoid poor local solutions.
- Use accessible language: "parameter exploration" instead of "hyperparameter search."
- Focus on practical outcomes and how to interpret the results.
</communication_style>

Acting as the TorchRDIT Design Assistant from, please generate a Python script using TorchRDIT to
optimize an optical metasurface operating at the wavelength of 5.2 um. The metasurface consists of
a grating layer on top and a substrate. The grating layer (650 nm) is a two-layer PbTe model (top
half layer: n_top=4.8; bottom half layer: n_bottom=5.4, k_bottom=0.01). The substrate is CaF2 (
n_caf2=1.4). The periodicity is 2.5 um in both the x and y directions. The incident light (TM mode,
 x-polarized) is transmitted from the substrate and out of the top grating layer to the air in the
normal direction. The grating layer is a rectangular pillar, and its length and width are to be
optimized by TorchRDIT to achieve a transmission efficiency greater than 80% while also meeting a
target transmitted TM phase of 170 degrees. The relative errors of phase in degrees should be less
than 5%. Use Context7 to search the docs of TorchRDIT and use torchrdit-mcp to get coding templates.
```

Listing 1: **P2: Prompt with workflow instructions and optimization strategies**

S2 Details of Performance Metrics and Error Type Definition

Table S1: Composite Score Classification

Score Range	Classification	Typical Design Characteristics
≥ 0.85	Excellent	High transmission ($\geq$90%) + accurate phase ($\leq$5°)
0.70–0.84	Good	Good transmission (85–90%) + acceptable phase (5–8°)
0.55–0.69	Acceptable	Threshold transmission (80–85%) + tolerable phase (8–12°)
< 0.55	Poor/Inadequate	Insufficient performance for practical application

Table S2: Performance Metrics Comparison Between Prompting Strategies

Strategy	Total Trials	Successful Trials	Trans $\geq$80% Count	Trans $\geq$80% %	Phase $\leq$8.5° Count	Phase $\leq$8.5° %	Both Requirements Count	Both Requirements %
P1	50	47	20	43%	28	60%	11	23%
P2	50	50	45	90%	41	82%	38	76%

Table S3: Top 6 Performing Designs Grouped by Strategy

Rank	Trial ID	Strategy	Composite Score	Classification	Dimensions (μm) x_length	Dimensions (μm) y_length	Trans. Eff. (%)	Phase Target (°)	Phase Error (°)	Turns	DES
Strategy P1											
1	P1-R06	P1	0.9841	Excellent	0.940	0.879	99.20	170.2	0.2	2	0.492
5	P1-R09	P1	0.9150	Excellent	2.255	2.094	93.20	170.0	0.0	2	0.458
6	P1-R41	P1	0.8938	Excellent	2.231	2.228	91.50	170.0	0.0	4	0.223
Strategy P2											
2	P2-R40	P2	0.9443	Excellent	0.756	1.969	95.54	170.0	0.0	1	0.944
3	P2-R18	P2	0.9440	Excellent	1.977	0.754	95.52	170.0	0.0	1	0.944
4	P2-R46	P2	0.9325	Excellent	2.027	2.201	94.60	170.0	0.0	2	0.466

Table S4: Categorization of Error Types Observed During LLM-MCP Inverse Design Trials

Error Type	Definition
API_HALLUCINATION	Inventing non-existent functions, methods, or parameters
RUNTIME_ERROR	Errors occurring during code execution
API_SELECTION	Choosing inappropriate functions for the task
GRADIENT_ERROR	Improper gradient handling or optimization approach
SOLVER_MISUNDERSTANDING	Fundamental misunderstanding of how the solver works
CONTEXT_LOSS	Forgetting or confusing previously established context
PROMPT_MISINTERPRETATION	Misunderstanding the user's requirements
SESSION_ABANDONED	User gave up due to repeated failures
SYNTAX_ERROR	Basic code syntax issues preventing execution
DOCUMENTATION_MISREADING	Incorrect reading or interpretation of documentation

S3 Examples of Generated Scripts for the Metasurfaces Inverse Design

S3.1 Script Generated by P1 (Natural Language): P1-R06

```python
"""
TorchRDIT Metasurface Optimization Script

This script optimizes a metasurface consisting of a grating layer on a substrate
to achieve specific transmission efficiency and phase targets.

Structure:
- Grating layer (650 nm): Two-layer PbTe model
  - Top half (325 nm): n_top = 4.8
  - Bottom half (325 nm): n_bottom = 5.4, k_bottom = 0.01
- Substrate: CaF2 (n_caf2 = 1.4)
- Periodicity: 2.5 um in both x and y directions
- Operating wavelength: 5.2 um
- Incident light: TM mode, x-polarized, normal incidence from substrate

Optimization Goals:
- Transmission efficiency > 80%
- Target transmitted TM phase: 170 degrees
- Phase error < 5%
- Optimize rectangular pillar length and width
"""

import numpy as np
import torch
import matplotlib.pyplot as plt
from tqdm import trange
import time

# TorchRDIT imports
from torchrdit.solver import get_solver_builder
from torchrdit.shapes import ShapeGenerator
from torchrdit.utils import create_material
from torchrdit.constants import Algorithm, Precision

# Physical constants and units
NM = 1e-3  # Convert nm to um
DEGREE = np.pi / 180  # Convert degrees to radians

# Device parameters
WAVELENGTH = 5.2  # um
PERIOD = 2.5      # um
GRATING_THICKNESS = 650 * NM  # Total grating thickness (um)
LAYER_THICKNESS = GRATING_THICKNESS / 2  # Each sub-layer thickness (um)

# Material properties
N_TOP = 4.8                    # Top layer refractive index
N_BOTTOM = 5.4                 # Bottom layer refractive index
K_BOTTOM = 0.01                # Bottom layer extinction coefficient
N_CAF2 = 1.4                   # CaF2 substrate refractive index
N_AIR = 1.0                    # Air

# Target specifications
TARGET_TRANSMISSION = 0.80     # 80% minimum transmission
TARGET_PHASE = 170 * DEGREE    # 170 degrees target phase
PHASE_TOLERANCE = 5 * DEGREE   # +-5 degrees phase error

def create_materials():
    """Create materials for the simulation."""

    # Air (surrounding medium)
    air = create_material(name='air', permittivity=N_AIR**2)

    # CaF2 substrate
    caf2 = create_material(name='caf2', permittivity=N_CAF2**2)

    # Top PbTe layer (real only)
    pbte_top = create_material(name='pbte_top', permittivity=N_TOP**2)

    # Bottom PbTe layer (complex)
    eps_bottom = complex(N_BOTTOM**2, 2*N_BOTTOM*K_BOTTOM)
    pbte_bottom = create_material(name='pbte_bottom', permittivity=eps_bottom)

    return [air, caf2, pbte_top, pbte_bottom]

def setup_solver():
    """Setup the TorchRDIT solver with proper configuration."""

    # Create solver builder
    builder = get_solver_builder()

    # Configure solver
    builder.with_algorithm(Algorithm.RDIT)  # Use R-DIT for better optimization
    builder.with_precision(Precision.DOUBLE)  # Double precision for accuracy
    builder.with_real_dimensions([512, 512])  # Real space resolution
    builder.with_k_dimensions([9, 9])  # Fourier harmonics (adjust as needed)
    builder.with_wavelengths(np.array([WAVELENGTH]))
```

```python
    builder.with_length_unit('um')

    # Define unit cell (square periodicity)
    t1 = torch.tensor([[PERIOD, 0.0]], dtype=torch.float64)
    t2 = torch.tensor([[0.0, PERIOD]], dtype=torch.float64)
    builder.with_lattice_vectors(t1, t2)

    # Use GPU if available
    device = 'cuda' if torch.cuda.is_available() else 'cpu'
    builder.with_device(device)
    print(f"Using device: {device}")

    # Set R-DIT order for stability
    builder.with_rdit_order(10)

    # Build solver
    solver = builder.build()

    # Add materials
    materials = create_materials()
    solver.add_materials(material_list=materials)

    return solver

def build_layer_stack(solver):
    """Build the layer stack for the metasurface."""

    # Layer stack order: substrate -> bottom PbTe -> top PbTe -> air
    # (reflection region -> ... -> transmission region)

    # Layer 0: CaF2 substrate (semi-infinite, reflection region)
    solver.add_layer(
        material_name='caf2',
        thickness=torch.tensor(0.0, dtype=torch.float64),  # Semi-infinite
        is_homogeneous=True
    )

    # Layer 1: Bottom PbTe layer (patterned)
    solver.add_layer(
        material_name='pbte_bottom',
        thickness=torch.tensor(LAYER_THICKNESS, dtype=torch.float64),
        is_homogeneous=False,  # Will be patterned
        is_optimize=True
    )

    # Layer 2: Top PbTe layer (patterned)
    solver.add_layer(
        material_name='pbte_top',
        thickness=torch.tensor(LAYER_THICKNESS, dtype=torch.float64),
        is_homogeneous=False,  # Will be patterned
        is_optimize=True
    )

    # Layer 3: Air (semi-infinite, transmission region)
    solver.add_layer(
        material_name='air',
        thickness=torch.tensor(0.0, dtype=torch.float64),  # Semi-infinite
        is_homogeneous=True
    )

    print("Layer stack built (substrate -> air):")
    print("  Layer 0: CaF2 substrate (semi-infinite, reflection region)")
    print(f"  Layer 1: Bottom PbTe ({LAYER_THICKNESS*1000:.1f} nm, patterned)")
    print(f"  Layer 2: Top PbTe ({LAYER_THICKNESS*1000:.1f} nm, patterned)")
    print("  Layer 3: Air (semi-infinite, transmission region)")

def create_source(solver):
    """Create the incident source (TM mode, x-polarized)."""

    # Configure reflection/transmission regions for substrate incidence
    # Reflection region = substrate (where light comes from)
    # Transmission region = air (where light goes to)
    solver.update_ref_material('caf2')  # Light comes from substrate
    solver.update_trn_material('air')   # Light goes to air

    # TM mode with x-polarization, normal incidence
    source = solver.add_source(
        theta=0.0,    # Normal incidence
        phi=0.0,      # Azimuthal angle
        pte=0.0,      # TE polarization component
        ptm=1.0       # TM polarization component (x-polarized)
    )

    return source

def create_rectangular_mask(solver, width, length):
    """Create rectangular pillar mask for the grating structure."""

    shapegen = ShapeGenerator.from_solver(solver)

    # Create rectangular mask centered at origin
    mask = shapegen.generate_rectangle_mask(
```

```
        center=[0.0, 0.0],
        width=width,
        height=length,
        soft_edge=0.01  # Small soft edge for gradient smoothness
    )

    return mask

def update_geometry(solver, width, length):
    """Update the geometry of both grating layers."""

    # Create rectangular mask
    mask = create_rectangular_mask(solver, width, length)

    # Apply same mask to both layers
    # Layer 1: Bottom PbTe layer
    solver.update_er_with_mask(
        mask=mask,
        layer_index=1,
        bg_material='air'
    )

    # Layer 2: Top PbTe layer
    solver.update_er_with_mask(
        mask=mask,
        layer_index=2,
        bg_material='air'
    )

def calculate_phase(result):
    """Calculate the transmitted phase from the complex transmission coefficient."""

    # Get complex transmission coefficient (zeroth order)
    # Note: TorchRDIT might not have get_order_transmission method
    # Try using the transmission field instead
    try:
        # Try to get complex transmission coefficient directly
        t_complex = result.transmission_field.x[0]  # X-component for TM x-polarized
        phase = torch.angle(t_complex[0, 0])  # Take center pixel
    except AttributeError:
        # Fallback: use transmission magnitude only (no phase info)
        print("Warning: Cannot extract phase information. Using zero phase.")
        phase = torch.tensor(0.0, dtype=torch.float64)

    return phase

def objective_function(solver, source, width, length):
    """
    Objective function for optimization.

    Combines transmission efficiency and phase targets with constraints.
    """

    # Update geometry
    update_geometry(solver, width, length)

    # Solve
    result = solver.solve(source)

    # Get transmission efficiency
    transmission = result.transmission[0]

    # Calculate transmitted phase
    phase = calculate_phase(result)

    # Phase loss (wrapped to [-pi, pi])
    phase_error = torch.abs(phase - TARGET_PHASE)
    phase_error = torch.min(phase_error, 2*np.pi - phase_error)  # Handle wrapping

    # Combined loss function
    # Transmission loss: penalize if below target
    trans_loss = torch.relu(TARGET_TRANSMISSION - transmission)

    # Phase loss: quadratic penalty
    phase_loss = (phase_error / PHASE_TOLERANCE)**2

    # Total loss with weighting
    total_loss = 10.0 * trans_loss + phase_loss

    return total_loss, transmission, phase

def optimize_metasurface():
    """Main optimization function."""

    print("=== TorchRDIT Metasurface Optimization ===\n")

    # Setup solver and geometry
    solver = setup_solver()
    build_layer_stack(solver)
    source = create_source(solver)

    # Initialize optimization parameters
```

```
    # Start with reasonable initial guesses
    width = torch.tensor(1.0, dtype=torch.float64, requires_grad=True)   # um
    length = torch.tensor(1.0, dtype=torch.float64, requires_grad=True)  # um

    # Setup optimizer
    optimizer = torch.optim.Adam([width, length], lr=0.02)
    scheduler = torch.optim.lr_scheduler.MultiStepLR(
        optimizer, milestones=[50, 100, 150], gamma=0.8
    )

    # Optimization tracking
    num_epochs = 200
    history = {
        ’loss’: [],
        ’transmission’: [],
        ’phase’: [],
        ’width’: [],
        ’length’: []
    }

    print("Starting optimization...")
    print(f"Target: T > {TARGET_TRANSMISSION:.1%}, Phase = {TARGET_PHASE/DEGREE:.1f} deg")
    print(f"Phase tolerance: +-{PHASE_TOLERANCE/DEGREE:.1f} deg\n")

    start_time = time.time()

    # Optimization loop
    for epoch in trange(num_epochs, desc="Optimizing"):

        optimizer.zero_grad()

        # Calculate loss
        loss, transmission, phase = objective_function(solver, source, width, length)

        # Backward pass
        loss.backward()

        # Update parameters
        optimizer.step()
        scheduler.step()

        # Apply physical constraints
        with torch.no_grad():
            width.clamp_(0.1, PERIOD - 0.1)   # Keep within unit cell
            length.clamp_(0.1, PERIOD - 0.1)  # Keep within unit cell

        # Record history
        history[’loss’].append(loss.item())
        history[’transmission’].append(transmission.item())
        history[’phase’].append(phase.item() / DEGREE)  # Convert to degrees
        history[’width’].append(width.item())
        history[’length’].append(length.item())

        # Print progress every 20 epochs
        if (epoch + 1) % 20 == 0:
            print(f"\\nEpoch {epoch+1:3d}: Loss = {loss.item():.4f}")
            print(f"  Transmission = {transmission.item():.1%}")
            print(f"  Phase = {phase.item()/DEGREE:.1f} deg (target: {TARGET_PHASE/DEGREE:.1f} deg)")
            print(f"  Width = {width.item():.3f} um, Length = {length.item():.3f} um")

    optimization_time = time.time() - start_time

    # Final evaluation
    print(f"\\n=== Optimization Complete ===")
    print(f"Time: {optimization_time:.1f} seconds")

    with torch.no_grad():
        final_loss, final_trans, final_phase = objective_function(
            solver, source, width, length
        )

    print(f"\\nFinal Results:")
    print(f"  Width = {width.item():.3f} um")
    print(f"  Length = {length.item():.3f} um")
    print(f"  Transmission = {final_trans.item():.1%}")
    print(f"  Phase = {final_phase.item()/DEGREE:.1f} deg")

    # Check if targets are met
    trans_met = final_trans.item() >= TARGET_TRANSMISSION
    phase_error_deg = abs(final_phase.item()/DEGREE - TARGET_PHASE/DEGREE)
    phase_met = phase_error_deg <= PHASE_TOLERANCE/DEGREE

    print(f"\\nTarget Achievement:")
    print(f"  Transmission > {TARGET_TRANSMISSION:.1%}: {’Yes’ if trans_met else ’No’}")
    print(f"  Phase error < {PHASE_TOLERANCE/DEGREE:.1f} deg: {’Yes’ if phase_met else ’No’} ({phase_error_deg:.1f} deg)")

    # Plot optimization history
    plot_optimization_history(history)

    return width.item(), length.item(), final_trans.item(), final_phase.item()/DEGREE

def plot_optimization_history(history):
```

```
    """Plot the optimization convergence history."""

    fig, ((ax1, ax2), (ax3, ax4)) = plt.subplots(2, 2, figsize=(12, 10))

    epochs = range(1, len(history['loss']) + 1)

    # Loss evolution
    ax1.semilogy(epochs, history['loss'])
    ax1.set_xlabel('Epoch')
    ax1.set_ylabel('Loss')
    ax1.set_title('Optimization Loss')
    ax1.grid(True)

    # Transmission evolution
    ax2.plot(epochs, [t*100 for t in history['transmission']], 'g-')
    ax2.axhline(y=TARGET_TRANSMISSION*100, color='r', linestyle='--',
                label=f'Target ({TARGET_TRANSMISSION:.0%})')
    ax2.set_xlabel('Epoch')
    ax2.set_ylabel('Transmission (%)')
    ax2.set_title('Transmission Efficiency')
    ax2.legend()
    ax2.grid(True)

    # Phase evolution
    ax3.plot(epochs, history['phase'], 'b-')
    ax3.axhline(y=TARGET_PHASE/DEGREE, color='r', linestyle='--',
                label=f'Target ({TARGET_PHASE/DEGREE:.0f} deg)')
    ax3.fill_between(epochs,
                     TARGET_PHASE/DEGREE - PHASE_TOLERANCE/DEGREE,
                     TARGET_PHASE/DEGREE + PHASE_TOLERANCE/DEGREE,
                     alpha=0.2, color='red', label='Tolerance')
    ax3.set_xlabel('Epoch')
    ax3.set_ylabel('Phase (degrees)')
    ax3.set_title('Transmitted Phase')
    ax3.legend()
    ax3.grid(True)

    # Geometry evolution
    ax4.plot(epochs, history['width'], 'b-', label='Width')
    ax4.plot(epochs, history['length'], 'r-', label='Length')
    ax4.axhline(y=PERIOD, color='k', linestyle=':', alpha=0.5, label='Period')
    ax4.set_xlabel('Epoch')
    ax4.set_ylabel('Dimension (um)')
    ax4.set_title('Pillar Dimensions')
    ax4.legend()
    ax4.grid(True)

    plt.tight_layout()
    plt.savefig('metasurface_optimization_history.png', dpi=300, bbox_inches='tight')
    plt.show()

def analyze_final_design(width, length):
    """Perform detailed analysis of the optimized design."""

    print(f"\\n=== Final Design Analysis ===")

    # Setup solver for analysis
    solver = setup_solver()
    build_layer_stack(solver)
    source = create_source(solver)

    # Set final geometry
    update_geometry(solver, width, length)

    # Solve for detailed results
    result = solver.solve(source)

    # Calculate all relevant metrics
    transmission = result.transmission[0].item()
    reflection = result.reflection[0].item()
    phase_rad = calculate_phase(result).item()
    phase_deg = phase_rad / DEGREE

    # Zero-order efficiencies
    zero_order_t = result.get_order_transmission_efficiency(0, 0)[0].item()
    zero_order_r = result.get_order_reflection_efficiency(0, 0)[0].item()

    print(f"\\nDetailed Results:")
    print(f"  Wavelength: {WAVELENGTH} um")
    print(f"  Pillar width: {width:.3f} um ({width/PERIOD:.1%} of period)")
    print(f"  Pillar length: {length:.3f} um ({length/PERIOD:.1%} of period)")
    print(f"  Fill factor: {(width*length)/(PERIOD**2):.1%}")
    print(f"\\nOptical Performance:")
    print(f"  Total transmission: {transmission:.1%}")
    print(f"  Total reflection: {reflection:.1%}")
    print(f"  Energy conservation: {transmission + reflection:.1%}")
    print(f"  Zero-order transmission: {zero_order_t:.1%}")
    print(f"  Zero-order reflection: {zero_order_r:.1%}")
    print(f"  Transmitted phase: {phase_deg:.1f} deg")
    print(f"  Phase error: {abs(phase_deg - TARGET_PHASE/DEGREE):.1f} deg")

if __name__ == "__main__":
```

```
    # Run optimization
    try:
        width_opt, length_opt, trans_final, phase_final = optimize_metasurface()

        # Detailed analysis
        analyze_final_design(width_opt, length_opt)

        print(f"\\n=== Summary ===")
        print(f"Optimized rectangular pillar:")
        print(f"  Width: {width_opt:.3f} um")
        print(f"  Length: {length_opt:.3f} um")
        print(f"  Transmission: {trans_final:.1%}")
        print(f"  Phase: {phase_final:.1f} deg")

    except Exception as e:
        print(f"Error during optimization: {e}")
        import traceback
        traceback.print_exc()
```

S3.2 Script Generated by P2 (Structured Guidance): P2-R40

```
#!/usr/bin/env python3
"""
TorchRDIT Metasurface Optimization Script

This script optimizes a rectangular pillar metasurface operating at 5.2 um wavelength.
The design consists of:
- Grating layer: 650 nm thick, two-layer PbTe model
  - Top half: n_top = 4.8
  - Bottom half: n_bottom = 5.4, k_bottom = 0.01
- Substrate: CaF2 (n = 1.4)
- Periodicity: 2.5 um x 2.5 um
- Incident: TM mode (x-polarized), normal incidence from substrate

Optimization goals:
- Transmission efficiency > 80%
- Target transmitted TM phase: 170 degrees
- Phase error < 5% (relative error)
- Optimize pillar length and width

Strategy: Two-stage global optimization
1. Parameter sweep to explore design space
2. Gradient-based refinement for best candidates
"""

# Basic imports for TorchRDIT
import numpy as np
import torch
import matplotlib.pyplot as plt
from torchrdit.solver import get_solver_builder
from torchrdit.shapes import ShapeGenerator
from torchrdit.utils import create_material
from torchrdit.constants import Algorithm, Precision
from torchrdit.viz import plot_layer, display_fitted_permittivity
import time
from tqdm import trange

# Define units (all calculations will use these units)
um = 1  # micrometers as base unit
nm = 1e-3 * um  # nanometers
mm = 1e3 * um  # millimeters
degrees = np.pi / 180  # convert degrees to radians

# Device specifications
wavelength = 5.2 * um  # Operating wavelength
period = 2.5 * um  # Periodicity in both x and y
grating_thickness = 650 * nm  # Total grating thickness
half_grating = grating_thickness / 2  # Half thickness for two-layer model

# Material properties
n_top = 4.8  # Top half of grating
n_bottom = 5.4  # Bottom half of grating
k_bottom = 0.01  # Imaginary part for bottom half
n_caf2 = 1.4  # CaF2 substrate
n_air = 1.0  # Air (transmission region)

# Optimization targets
target_transmission = 0.80  # Minimum 80% transmission
target_phase_deg = 170  # Target phase in degrees
target_phase_rad = target_phase_deg * degrees
phase_error_tolerance = 0.05  # 5% relative error

print("=" * 60)
print("TorchRDIT Metasurface Optimization")
print("=" * 60)
print(f"Wavelength: {wavelength*1e3:.1f} nm")
print(f"Period: {period*1e3:.1f} nm")
```

```python
print(f"Target transmission: {target_transmission*100:.0f}%")
print(f"Target phase: {target_phase_deg:.0f} deg")
print("=" * 60)

def create_solver(wavelength, period):
    """Create TorchRDIT solver with proper configuration."""

    # Create solver using builder pattern
    builder = get_solver_builder()

    # Essential configuration
    builder.with_algorithm(Algorithm.RCWA)  # Use RCWA for this application
    builder.with_precision(Precision.DOUBLE)  # Double precision for accuracy
    builder.with_real_dimensions([512, 512])  # Real space grid resolution
    builder.with_k_dimensions([9, 9])  # Fourier space harmonics (adequate for this structure)
    builder.with_wavelengths(np.array([wavelength]))  # Single wavelength
    builder.with_length_unit('um')

    # Define lattice vectors for periodic boundary conditions
    t1 = torch.tensor([[period, 0]])
    t2 = torch.tensor([[0, period]])
    builder.with_lattice_vectors(t1, t2)

    # Set computation device
    device = 'cuda' if torch.cuda.is_available() else 'cpu'
    builder.with_device(device)
    print(f"Using device: {device}")

    # Build the solver
    solver = builder.build()

    return solver

def create_materials():
    """Create all materials needed for the simulation."""

    # Create materials using permittivity (n^2 for lossless, complex for lossy)
    air = create_material(name='air', permittivity=n_air**2)
    caf2 = create_material(name='caf2', permittivity=n_caf2**2)
    pbte_top = create_material(name='pbte_top', permittivity=n_top**2)

    # Bottom PbTe with loss: epsilon_r = (n + ik)^2 = n^2 - k^2 + 2ink
    n_complex = n_bottom + 1j * k_bottom
    pbte_bottom = create_material(name='pbte_bottom', permittivity=n_complex**2)

    materials = [air, caf2, pbte_top, pbte_bottom]

    print("Materials created:")
    print(f"  Air: epsilon_r = {n_air**2:.1f}")
    print(f"  CaF2: epsilon_r = {n_caf2**2:.1f}")
    print(f"  PbTe (top): epsilon_r = {n_top**2:.1f}")
    print(f"  PbTe (bottom): epsilon_r = {n_complex**2:.3f}")

    return materials

def setup_layer_stack(solver, materials):
    """Setup the layer stack: substrate -> bottom grating -> top grating -> air."""

    # Add materials to solver
    solver.add_materials(material_list=materials)

    # Set reference material (incident medium - substrate)
    solver.update_ref_material('caf2')

    # Set transmission material (air)
    solver.update_trn_material('air')

    # Add layers from bottom to top
    # Layer 0: Bottom half of grating (PbTe with loss)
    solver.add_layer(
        material_name='pbte_bottom',
        thickness=torch.tensor(half_grating, dtype=torch.float64),
        is_homogeneous=False,  # Make it patternable
        is_optimize=True
    )

    # Layer 1: Top half of grating (PbTe without loss)
    solver.add_layer(
        material_name='pbte_top',
        thickness=torch.tensor(half_grating, dtype=torch.float64),
        is_homogeneous=False,  # Make it patternable
        is_optimize=True
    )

    print("Layer stack configured:")
    print("  Incident medium: CaF2 substrate")
    print(f"  Layer 0: PbTe bottom ({half_grating*1e3:.0f} nm)")
    print(f"  Layer 1: PbTe top ({half_grating*1e3:.0f} nm)")
    print("  Transmission medium: Air")

def create_rectangular_mask(solver, width, height):
    """Create rectangular pillar mask for both grating layers."""
```

```python

    # Create shape generator
    shapegen = ShapeGenerator.from_solver(solver)

    # Generate rectangular mask centered at origin
    mask = shapegen.generate_rectangle_mask(
        center=[0.0, 0.0],  # Center at origin
        width=width,        # Width in um
        height=height,      # Height in um
        angle=0,           # No rotation
        soft_edge=0.001    # Small soft edge for numerical stability
    )

    return mask

def update_device_geometry(solver, width, height):
    """Update the device geometry with new pillar dimensions."""

    # Create mask for the rectangular pillar
    mask = create_rectangular_mask(solver, width, height)

    # Apply mask to both grating layers
    # Layer 0: Bottom grating layer
    solver.update_er_with_mask(
        mask=mask,
        layer_index=0,
        bg_material='air'  # Background is air (holes)
    )

    # Layer 1: Top grating layer
    solver.update_er_with_mask(
        mask=mask,
        layer_index=1,
        bg_material='air'  # Background is air (holes)
    )

def setup_source(solver):
    """Setup TM-polarized source (x-polarized) at normal incidence."""

    # TM mode: ptm=1.0, pte=0.0 for x-polarized light
    source = solver.add_source(
        theta=0 * degrees,  # Normal incidence
        phi=0 * degrees,    # Azimuthal angle
        pte=0.0,           # TE polarization amplitude (zero for TM)
        ptm=1.0            # TM polarization amplitude (x-polarized)
    )

    print("Source configured: TM mode (x-polarized), normal incidence")
    return source

def simulate_device(solver, source, width, height):
    """Simulate device with given pillar dimensions and return results."""

    # Update geometry
    update_device_geometry(solver, width, height)

    # Solve electromagnetic problem
    result = solver.solve(source)

    # Get zero-order transmission field components
    tx, ty, tz = result.get_zero_order_transmission()

    # Calculate transmission efficiency and phase
    transmission = result.transmission[0].item()
    phase_x = torch.angle(tx[0]).item()  # Phase in radians

    return transmission, phase_x, result

def calculate_objective(transmission, phase_x, target_transmission, target_phase_rad):
    """Calculate multi-objective loss function."""

    # Transmission loss (penalize if below target)
    trans_loss = max(0, target_transmission - transmission)**2

    # Phase loss (squared difference from target)
    phase_diff = phase_x - target_phase_rad
    # Normalize phase difference to [-pi, pi]
    while phase_diff > np.pi:
        phase_diff -= 2 * np.pi
    while phase_diff < -np.pi:
        phase_diff += 2 * np.pi
    phase_loss = phase_diff**2

    # Combined loss (weight phase more heavily if transmission is adequate)
    if transmission >= target_transmission:
        total_loss = 0.1 * trans_loss + phase_loss
    else:
        total_loss = 10 * trans_loss + phase_loss

    return total_loss

def parameter_sweep(solver, source):
```

```python
    """Stage 1: Parameter sweep to explore design space."""

    print("\nStage 1: Parameter Exploration")
    print("-" * 30)

    # Define parameter ranges (in micrometers)
    widths = np.linspace(0.5, 2.0, 10)  # 0.5 to 2.0 um
    heights = np.linspace(0.5, 2.0, 10)  # 0.5 to 2.0 um

    results = []
    best_loss = float('inf')
    best_params = None

    print(f"Evaluating {len(widths) * len(heights)} parameter combinations...")

    for i, width in enumerate(widths):
        for j, height in enumerate(heights):
            try:
                # Simulate device
                transmission, phase_x, _ = simulate_device(solver, source, width, height)

                # Calculate objective
                loss = calculate_objective(transmission, phase_x, target_transmission, target_phase_rad)

                # Convert phase to degrees for display
                phase_deg = phase_x * 180 / np.pi

                results.append({
                    'width': width,
                    'height': height,
                    'transmission': transmission,
                    'phase_deg': phase_deg,
                    'loss': loss
                })

                # Track best result
                if loss < best_loss:
                    best_loss = loss
                    best_params = (width, height)

                # Print progress occasionally
                if (i * len(heights) + j + 1) % 20 == 0:
                    print(f"  Progress: {i * len(heights) + j + 1}/{len(widths) * len(heights)}")

            except Exception as e:
                print(f"  Error at width={width:.2f}, height={height:.2f}: {e}")
                continue

    # Sort results by loss
    results.sort(key=lambda x: x['loss'])

    print(f"\nParameter sweep completed. Best parameters:")
    print(f"  Width: {best_params[0]:.3f} um")
    print(f"  Height: {best_params[1]:.3f} um")
    print(f"  Loss: {best_loss:.6f}")

    # Return top 5 candidates for gradient optimization
    return results[:5]

def gradient_optimization(solver, source, initial_width, initial_height, num_epochs=200):
    """Stage 2: Gradient-based optimization starting from given initial point."""

    # Define optimizable parameters
    width_param = torch.tensor(initial_width, requires_grad=True, dtype=torch.float64)
    height_param = torch.tensor(initial_height, requires_grad=True, dtype=torch.float64)

    # Setup optimizer
    params = [width_param, height_param]
    optimizer = torch.optim.Adam(params, lr=0.01)

    # Track best result
    best_loss = float('inf')
    best_width = initial_width
    best_height = initial_height
    best_transmission = 0
    best_phase = 0

    print(f"\nGradient optimization from width={initial_width:.3f}, height={initial_height:.3f}")
    print("Epoch | Width   | Height  | Trans% | Phase deg | Loss")
    print("-" * 55)

    for epoch in range(num_epochs):
        optimizer.zero_grad()

        try:
            # Current parameter values
            w = width_param.item()
            h = height_param.item()

            # Create mask (must be done in forward pass for gradients)
            mask = create_rectangular_mask(solver, width_param, height_param)

```

```python
            # Update layers with mask
            solver.update_er_with_mask(mask=mask, layer_index=0, bg_material='air')
            solver.update_er_with_mask(mask=mask, layer_index=1, bg_material='air')

            # Solve
            result = solver.solve(source)

            # Get transmission and phase
            tx, ty, tz = result.get_zero_order_transmission()
            transmission = result.transmission[0]
            phase_x = torch.angle(tx[0])

            # Calculate loss
            trans_loss = torch.max(torch.tensor(0.0), target_transmission - transmission)**2

            phase_diff = phase_x - target_phase_rad
            # Normalize phase difference
            phase_diff = torch.remainder(phase_diff + np.pi, 2*np.pi) - np.pi
            phase_loss = phase_diff**2

            # Combined loss
            if transmission.item() >= target_transmission:
                total_loss = 0.1 * trans_loss + phase_loss
            else:
                total_loss = 10 * trans_loss + phase_loss

            # Backward pass
            total_loss.backward()

            # Apply gradient clipping for stability
            torch.nn.utils.clip_grad_norm_(params, max_norm=1.0)

            # Update parameters
            optimizer.step()

            # Apply physical constraints
            with torch.no_grad():
                width_param.clamp_(0.1, 2.4)  # Keep within reasonable bounds
                height_param.clamp_(0.1, 2.4)

            # Track best result
            current_loss = total_loss.item()
            if current_loss < best_loss:
                best_loss = current_loss
                best_width = w
                best_height = h
                best_transmission = transmission.item()
                best_phase = phase_x.item() * 180 / np.pi

            # Print progress
            if epoch % 20 == 0 or epoch == num_epochs - 1:
                print(f"{epoch:5d} | {w:7.3f} | {h:7.3f} | {transmission.item()*100:6.1f} | "
                      f"{phase_x.item()*180/np.pi:6.1f} | {current_loss:.6f}")

        except Exception as e:
            print(f"  Error at epoch {epoch}: {e}")
            break

    return best_width, best_height, best_transmission, best_phase, best_loss

def main():
    """Main optimization workflow."""

    # Create solver and setup
    solver = create_solver(wavelength, period)
    materials = create_materials()
    setup_layer_stack(solver, materials)
    source = setup_source(solver)

    # Stage 1: Parameter sweep
    candidates = parameter_sweep(solver, source)

    if not candidates:
        print("No valid candidates found in parameter sweep!")
        return

    print(f"\nTop 5 candidates from parameter sweep:")
    for i, candidate in enumerate(candidates):
        print(f"  {i+1}. W={candidate['width']:.3f}, H={candidate['height']:.3f}, "
              f"T={candidate['transmission']*100:.1f}%, phi={candidate['phase_deg']:.1f} deg, "
              f"Loss={candidate['loss']:.6f}")

    # Stage 2: Gradient optimization on top candidates
    print("\nStage 2: Gradient-based Refinement")
    print("-" * 40)

    best_overall_loss = float('inf')
    best_design = None

    for i, candidate in enumerate(candidates):
        print(f"\nOptimizing candidate {i+1}/5...")
```

```python
        try:
            opt_width, opt_height, opt_trans, opt_phase, opt_loss = gradient_optimization(
                solver, source, candidate['width'], candidate['height'], num_epochs=100
            )

            if opt_loss < best_overall_loss:
                best_overall_loss = opt_loss
                best_design = {
                    'width': opt_width,
                    'height': opt_height,
                    'transmission': opt_trans,
                    'phase_deg': opt_phase,
                    'loss': opt_loss
                }

        except Exception as e:
            print(f"  Optimization failed for candidate {i+1}: {e}")
            continue

    # Final results
    print("\n" + "=" * 60)
    print("OPTIMIZATION RESULTS")
    print("=" * 60)

    if best_design:
        print(f"Optimal pillar dimensions:")
        print(f"  Width:  {best_design['width']:.3f} um ({best_design['width']*1000:.0f} nm)")
        print(f"  Height: {best_design['height']:.3f} um ({best_design['height']*1000:.0f} nm)")
        print(f"\nPerformance:")
        print(f"  Transmission: {best_design['transmission']*100:.2f}%")
        print(f"  Phase: {best_design['phase_deg']:.1f} deg")
        print(f"  Loss: {best_design['loss']:.6f}")

        # Check if goals are met
        trans_ok = best_design['transmission'] >= target_transmission
        phase_error = abs(best_design['phase_deg'] - target_phase_deg) / target_phase_deg
        phase_ok = phase_error <= phase_error_tolerance

        print(f"\nGoals assessment:")
        print(f"  Transmission > 80%: {'Yes' if trans_ok else 'No'}")
        print(f"  Phase error < 5%: {'Yes' if phase_ok else 'No'} ({phase_error*100:.1f}%)")

        if trans_ok and phase_ok:
            print(f"\nSUCCESS: All optimization goals achieved!")
        else:
            print(f"\n  Some goals not fully achieved. Consider:")
            if not trans_ok:
                print(f"    - Adjusting material properties or layer thickness")
            if not phase_ok:
                print(f"    - Fine-tuning phase target or increasing optimization epochs")
    else:
        print("X Optimization failed - no valid design found")
        print("Consider:")
        print("  - Expanding parameter search range")
        print("  - Adjusting material properties")
        print("  - Modifying layer structure")

    print("=" * 60)

if __name__ == "__main__":
    main()
```

S4 Code Snippets of TorchRDIT MCP Template

```
# Core templates - instructive and clear
TEMPLATES = {
    "basic_imports": """# Basic imports for TorchRDIT
import numpy as np
import torch
import matplotlib.pyplot as plt
from torchrdit.solver import get_solver_builder
from torchrdit.shapes import ShapeGenerator
from torchrdit.utils import create_material
from torchrdit.constants import Algorithm, Precision
from torchrdit.viz import plot_layer, display_fitted_permittivity
""",

    "unit_setup": """# Define units (all calculations will use these units)
um = 1  # micrometers as base unit
nm = 1e-3 * um  # nanometers
mm = 1e3 * um  # millimeters
degrees = np.pi / 180  # convert degrees to radians
""",

    "solver_setup": """# Create solver using builder pattern
builder = get_solver_builder()

# Essential configuration
builder.with_algorithm(Algorithm.RCWA)  # or Algorithm.RDIT
builder.with_precision(Precision.DOUBLE)  # SINGLE or DOUBLE
builder.with_real_dimensions([512, 512])  # Real space grid resolution
builder.with_k_dimensions([9, 9])  # Fourier space harmonics
builder.with_wavelengths(np.array([1.55]))  # Wavelengths in um
builder.with_length_unit(’um’)

# For periodic structures, define lattice vectors
period = 0.5  # um
t1 = torch.tensor([[period, 0]])
t2 = torch.tensor([[0, period]])
builder.with_lattice_vectors(t1, t2)

# Optional: Set computation device
builder.with_device(’cuda’ if torch.cuda.is_available() else ’cpu’)

# For RDIT algorithm only: set order (typically 8-15)
# builder.with_rdit_order(10)

# Build the solver
solver = builder.build()
""",

    "material_creation": """# Create materials

# Method 1: Using permittivity directly
air = create_material(name=’air’, permittivity=1.0)
silicon = create_material(name=’silicon’, permittivity=11.7)  # at 1.55um

# Method 2: Using refractive index (n)
# permittivity = n^2
glass = create_material(name=’glass’, permittivity=1.5**2)

# Method 3: Complex permittivity (for lossy materials)
gold = create_material(name=’gold’, permittivity=complex(-100, 10))

# Method 4: Dispersive material from file
# dispersive_si = create_material(
#     name=’silicon_dispersive’,
#     dielectric_dispersion=True,
#     user_dielectric_file=’Si_data.txt’,
#     data_format=’wl-eps’,  # wavelength-permittivity format
#     data_unit=’um’
# )

# Add materials to solver
solver.add_materials(material_list=[air, silicon])
# Add other materials as needed
""",

    "layer_stack": """# IMPORTANT: Layer Stack Structure
# The layer ordering in TorchRDIT is:
#
# TOP (Transmission side): trn_material
#             |
#         Layer N-1
#             |
#            ...
#             |
#         Layer 1
#             |
#         Layer 0
#             |
# BOTTOM (Reflection/Incident side): ref_material
#
```

```
# Light is incident from the bottom (ref_material side)

# Set the bottom material (where light comes from)
solver.update_ref_material('air')  # or your substrate material

# Add layers from bottom to top
solver.add_layer(
    material_name='silicon',
    thickness=torch.tensor(0.22, dtype=torch.float64),  # in um
    is_homogeneous=True  # False for patterned layers
)

# Add more layers as needed...

# Set the top material
solver.update_trn_material('air')  # or your superstrate material

# NOTE: There is NO 'inc_material' or 'update_inc_material' in TorchRDIT!
# The incident medium is the ref_material (reflection/bottom layer)
""",

    "patterned_layer": """# Creating patterned (non-homogeneous) layers

# First, add a non-homogeneous layer
solver.add_layer(
    material_name='foreground_material',  # foreground material, the name of the material should be added to the solver materials list first
    thickness=torch.tensor(0.5, dtype=torch.float64),
    is_homogeneous=False,  # This makes it patternable
    is_optimize=True  # Optional: mark for optimization
)

# Create shape generator
shapegen = ShapeGenerator.from_solver(solver)

# Generate shapes (example: circle)
mask = shapegen.generate_circle_mask(
    center=[0.0, 0.0],  # Center position
    radius=0.3,  # Radius in um
    soft_edge=0.001  # Soft edge width (0 for hard edge)
)

# Apply mask to the layer (layer_index starts from 0)
solver.update_er_with_mask(mask=mask,
                           layer_index=0,
                           bg_material='air', # background material if other than air (optional)
                           )

# For multiple shapes, combine them:
# mask1 = shapegen.generate_circle_mask(...)
# mask2 = shapegen.generate_rectangle_mask(...)
# combined = shapegen.combine_masks(mask1, mask2, operation='union')
""",

    "source_setup": """# Define the light source

# Create source with specified properties
source = solver.add_source(
    theta=0 * degrees,  # Incident angle from normal
    phi=0 * degrees,    # Azimuthal angle
    pte=1.0,           # TE polarization amplitude
    ptm=0.0            # TM polarization amplitude
)

# For angled incidence:
# source = solver.add_source(theta=30*degrees, phi=0, pte=1, ptm=0)

# For unpolarized light (equal TE and TM):
# source = solver.add_source(theta=0, phi=0, pte=1/np.sqrt(2), ptm=1/np.sqrt(2))
""",

    "solve_and_analyze": """# Solve the electromagnetic problem
result = solver.solve(source)

# Access simulation results
# Overall efficiencies (summed over all diffraction orders)
transmission_total = result.transmission  # Shape: (n_wavelengths,)
reflection_total = result.reflection    # Shape: (n_wavelengths,)

# Print results
for i, wavelength in enumerate(solver.lam0):
    print(f"lambda = {wavelength*1000:.1f} nm:")
    print(f"  Transmission: {transmission_total[i]*100:.2f}%")
    print(f"  Reflection: {reflection_total[i]*100:.2f}%")
    print(f"  Absorption: {(1-transmission_total[i]-reflection_total[i])*100:.2f}%")

# Access field components (zero order)
tx, ty, tz = result.get_zero_order_transmission()
rx, ry, rz = result.get_zero_order_reflection()

# Calculate phase
phase_t = torch.angle(tx[0])  # Phase of x-component, first wavelength
amplitude_t = torch.abs(tx[0])  # Amplitude
```

```

# Access diffraction efficiencies for specific orders
# efficiency = result.get_order_transmission_efficiency(order_x=1, order_y=0)
""",

    "optimization_basic": """# Basic optimization setup for inverse design

# Define parameter to optimize (example: circle radius)
radius = torch.tensor(0.2, requires_grad=True)  # Initial value

# Define objective function
def objective(solver, source, radius):
    # Update device geometry
    mask = shapegen.generate_circle_mask(center=[0, 0], radius=radius)
    solver.update_er_with_mask(mask=mask, layer_index=0)

    # Solve
    result = solver.solve(source)

    # Define loss (example: maximize transmission at first wavelength)
    loss = -result.transmission[0]  # Negative because we minimize

    return loss

# Setup optimizer
optimizer = torch.optim.Adam([radius], lr=0.01)

# Training loop
num_epochs = 100
for epoch in range(num_epochs):
    optimizer.zero_grad()
    loss = objective(solver, source, radius)
    loss.backward()
    optimizer.step()

    # Optional: Apply constraints
    with torch.no_grad():
        radius.data = torch.clamp(radius.data, 0.05, 0.45)

    if epoch % 10 == 0:
        print(f"Epoch {epoch}: Loss = {loss.item():.6f}, Radius = {radius.item():.3f}")
""",

    "shape_operations": """# Available shape generation functions

# Circle
circle = shapegen.generate_circle_mask(
    center=[x, y],
    radius=r,
    soft_edge=0.001
)

# Rectangle
rectangle = shapegen.generate_rectangle_mask(
    center=[x, y],
    width=w,
    height=h,
    angle=theta,  # Rotation angle in degrees
    soft_edge=0.001
)

# Polygon
vertices = [[x1, y1], [x2, y2], [x3, y3]]  # List of vertices
polygon = shapegen.generate_polygon_mask(
    polygon_points=vertices,
    center=[x, y],
    angle=theta,
    soft_edge=0.001
)

# Combine shapes using boolean operations
union = shapegen.combine_masks(mask1, mask2, operation='union')
intersection = shapegen.combine_masks(mask1, mask2, operation='intersection')
difference = shapegen.combine_masks(mask1, mask2, operation='difference')
xor = shapegen.combine_masks(mask1, mask2, operation='subtract')
""",

    "visualization": """# Visualization functions

# Plot layer structure (for patterned layers)
fig, ax = plt.subplots(figsize=(6, 6))
plot_layer(solver, layer_index=0, fig_ax=ax,
           title='Layer Structure',
           labels=('x (um)', 'y (um)'))
plt.show()

# For dispersive materials, plot fitted permittivity
# display_fitted_permittivity(solver, fig_ax=ax)

# Custom visualization of results
wavelengths_nm = solver.lam0 * 1000  # Convert to nm
plt.figure(figsize=(8, 6))
```

```
plt.plot(wavelengths_nm, result.transmission * 100, ’b-’, label=’Transmission’)
plt.plot(wavelengths_nm, result.reflection * 100, ’r-’, label=’Reflection’)
plt.xlabel(’Wavelength (nm)’)
plt.ylabel(’Efficiency (%)’)
plt.legend()
plt.grid(True)
plt.show()
""",

    "common_patterns": """# Common Design Patterns

# 1. Multilayer Stack (Bragg reflector example)
n_periods = 10
for i in range(n_periods):
    solver.add_layer(material_name=’high_index’, thickness=torch.tensor(d1))
    solver.add_layer(material_name=’low_index’, thickness=torch.tensor(d2))

# 2. Metasurface with periodic array
# Set up lattice vectors for periodic boundary conditions
period = 0.5  # um
t1 = torch.tensor([[period, 0]])
t2 = torch.tensor([[0, period]])
builder.with_lattice_vectors(t1, t2)

# 3. Parameter sweep
thicknesses = np.linspace(0.1, 0.5, 50)
transmissions = []
for t in thicknesses:
    solver_temp = create_new_solver_with_thickness(t)
    result = solver_temp.solve(source)
    transmissions.append(result.transmission[0].item())

# 4. Multi-wavelength optimization
# Define wavelengths spanning your range of interest
wavelengths = np.linspace(1.4, 1.7, 10)  # um
builder.with_wavelengths(wavelengths)
"""
}

# Optimization-specific templates
OPTIMIZATION_TEMPLATES = {
    "gradient_based": """# Gradient-based optimization workflow

# 1. Define optimizable parameters
params = {
    ’radius’: torch.tensor(0.2, requires_grad=True),
    ’thickness’: torch.tensor(0.3, requires_grad=True)
}

# 2. Define objective function
def objective_function(solver, source, params):
    # Update geometry based on parameters
    update_device_with_params(solver, params)

    # Solve
    result = solver.solve(source)

    # Calculate loss (example: target specific transmission)
    target_transmission = 0.95
    loss = (result.transmission[0] - target_transmission)**2

    return loss

# 3. Optimization loop with Adam
optimizer = torch.optim.Adam(params.values(), lr=0.01)

for epoch in range(200):
    optimizer.zero_grad()
    loss = objective_function(solver, source, params)
    loss.backward()
    optimizer.step()

    # Apply physical constraints
    with torch.no_grad():
        params[’radius’].clamp_(0.05, 0.45)
        params[’thickness’].clamp_(0.1, 1.0)
""",

    "multi_objective": """# Multi-objective optimization

def multi_objective(solver, source, params):
    result = solver.solve(source)

    # Multiple objectives
    obj1 = (result.transmission[0] - 0.9)**2  # Target 90% at lambda1
    obj2 = (result.transmission[1] - 0.1)**2  # Target 10% at lambda2
    obj3 = torch.abs(result.reflection[0] - 0.05)  # Target 5% reflection

    # Weighted sum
    weights = [1.0, 1.0, 0.5]
    total_loss = weights[0]*obj1 + weights[1]*obj2 + weights[2]*obj3

```

```
    return total_loss

# Or use separate losses for monitoring
losses = {'transmission': obj1, 'blocking': obj2, 'reflection': obj3}
"""
}

# API clarification templates
API_CLARIFICATIONS = {
    "layer_order": """# CRITICAL: Understanding Layer Order in TorchRDIT

The layer stack in TorchRDIT follows this structure:

    | z-direction (upward)
    |
    |  Transmission region (top) - semi-infinite
    |  ================================
    |  Layer N-1 (last added layer)
    |  --------------------------------
    |  Layer N-2
    |  --------------------------------
    |  ...
    |  --------------------------------
    |  Layer 1 (second added layer)
    |  --------------------------------
    |  Layer 0 (first added layer)
    |  ================================
    |  Reflection region (bottom) - semi-infinite
    |
    | Light incident from here

Key points:
1. Light is incident from the BOTTOM (reflection region)
2. The reflection region is also the incident medium
3. Layers are numbered in the order they are added (0, 1, 2, ...)
4. NO 'incident' material - use 'ref_material' for the incident medium
""",

    "material_api": """# Material-related API clarification

CORRECT API calls:
- solver.update_ref_material('material_name')  # Set bottom/incident material
- solver.update_trn_material('material_name')  # Set top material
- builder.with_ref_material(material_object)   # During building
- builder.with_trn_material(material_object)   # During building

INCORRECT (these don't exist):
- solver.update_inc_material()  N
- builder.with_inc_material()   N
- solver.set_incident_material() N

Remember: The incident medium is the ref_material (reflection/bottom layer)
""",

    "common_mistakes": """# Common Mistakes to Avoid

1. Layer ordering confusion:
   N Adding layers from top to bottom
   Y Add layers from bottom to top

2. Incident material:
   N solver.update_inc_material('air')
   Y solver.update_ref_material('air')

3. Forgetting material setup:
   N Using material names without adding materials first
   Y Always add materials before referencing them

4. Layer indexing:
   N Assuming layer 0 is at the top
   Y Layer 0 is the first layer added (bottom-most)

5. Gradient tracking:
   N Not setting requires_grad=True for optimizable parameters
   Y Always enable gradients for parameters you want to optimize
"""
}
```

S5 Overview of the TorchRDIT Design Workflow

S5.1 Solver and Formulation

`TorchRDIT` is a Fourier modal method (FMM) solver with the automatic differentiation capability [1, 2]. It implements both rigorous coupled-wave analysis (RCWA) and an eigendecomposition-free rigorous diffraction interface theory (R-DIT) for periodic layered photonic structures. Each physical layer i is represented by a scattering matrix S_i that relates the incident and outgoing modal coefficients. Layers are cascaded using the Redheffer star product, with "gap" (buffer) layers used to ensure compatibility with semi-analytical methods.

The formulation starts from the P–Q block form of Maxwell's equations in the Fourier domain,

$$\frac{\mathrm{d}}{\mathrm{d}\tilde{z}}\begin{bmatrix}\vec{\mathcal{E}}_{x,y}\\ \vec{\mathcal{H}}_{x,y}\end{bmatrix} = \begin{bmatrix}0 & \hat{\mathbb{P}}\\ \hat{\mathbb{Q}} & 0\end{bmatrix}\begin{bmatrix}\vec{\mathcal{E}}_{x,y}\\ \vec{\mathcal{H}}_{x,y}\end{bmatrix}, \quad \vec{\mathcal{E}}_{x,y} = \begin{bmatrix}\vec{\mathcal{E}}_{x}\\ \vec{\mathcal{E}}_{y}\end{bmatrix}, \quad \vec{\mathcal{H}}_{x,y} = \begin{bmatrix}\vec{\mathcal{H}}_{x}\\ \vec{\mathcal{H}}_{y}\end{bmatrix}, \tag{S5.1}$$

where $\hat{P}$ and $\hat{Q}$ are built from Toeplitz permittivity/permeability matrices and diagonal k-space operators, $\vec{\mathcal{E}}_{x,y}$, $\vec{\mathcal{H}}_{x,y}$ are the Fourier components of the tangential electric/magnetic field. In RCWA, the propagation within a layer is obtained through an eigen-decomposition of the modal operator, whereas R-DIT bypasses eigen-decomposition by expanding tangential fields from the layer center to the two interfaces using Taylor series. This yields closed-form expressions for the layer scattering matrix that involve only matrix multiplications and inversions. As implemented in PyTorch, all operations participate in automatic differentiation, so gradients of user-defined objectives with respect to geometry and material parameters are obtained through the solver without hand-derived adjoints; the matrix-only computations also map efficiently to graphics processing units (GPUs). This strictly physics-based, differentiable solving is fundamentally different from neural-network surrogates.

S5.2 Automatic differentiation and parameterization

`TorchRDIT` is implemented on PyTorch, which makes the complete Maxwell solve and objective evaluation a single computation graph. Design variables, including geometry masks, feature sizes, material constants, and layer thicknesses, are represented as tensors. For a chosen set of wavelength and angle samples, the solver returns complex transmission and reflection quantities (with order resolution when requested). User-defined metrics are computed from these quantities and combined into a scalar objective $\mathcal{L}(\theta)$. PyTorch autograd then provides $\partial\mathcal{L}/\partial\theta$ directly through the solver, so no hand-derived adjoints are required.

The parameterization is flexible. Common choices include: (i) differentiable masks for pixel, spline, or implicit representations, (ii) explicit shape variables, and (iii) material or thickness variables. All options are compatible with RCWA and R-DIT. The matrix-only computations in R-DIT and the spectral-domain structure are efficiently mapped to GPUs. This remains a strictly physics-based Maxwell solver rather than a learned surrogate.

S5.3 Objectives and optimization workflow

`TorchRDIT` reports complex field coefficients, with optional order resolution and polarization-resolved fields. From these quantities, users may define differentiable scalar objectives with respect to geometry and material parameters using PyTorch autograd. This enables gradient-based end-to-end inverse design directly inside the solver.

Let θ denote the design parameters, and let $\mathcal{S}$ be the set of wavelength and angle samples. For each $s \in \mathcal{S}$, let $m_k(\theta; s)$ be a metric computed by `TorchRDIT` (for example, total or order-specific transmission or reflection, polarization measures, or phase). A general objective can be written as

$$\mathcal{L}(\theta) = \sum_{s\in\mathcal{S}}\sum_{k=1}^{K} w_{s,k}\,\phi_k\big(m_k(\theta; s),\, \tau_k(s)\big) \;+\; \mathcal{P}(\theta), \tag{S5.2}$$

where $\tau_k(s)$ are targets; ϕ_k are per-metric penalties; $w_{s,k}$ are nonnegative weights; $\mathcal{P}(\theta)$ collects bounds and regularization. In the main text, the metrics used are transmission efficiency and transmitted phase aggregated over the designated samples.

The general optimization flow is shown in Algorithm (1). After configuring the periodic cell and layer stack and choosing a parameterization for geometry and material variables, we define the objective in (S5.2). Each iteration updates the stack from the current θ, solves the Maxwell equations over the selected wavelength/angle samples, evaluates $\mathcal{L}$, obtains $\partial\mathcal{L}/\partial\theta$ via autograd, and applies a generic gradient-based update with optional bound enforcement and filtering. This workflow applies to both RCWA and R-DIT in `TorchRDIT`.

Algorithm 1 Gradient-based inverse design with `TorchRDIT`

Input: sample set $\mathcal{S}$ (wavelength, angle), initial parameters θ, weights $\{w_s\}$, tolerance ε
Build and configure `TorchRDIT` (lattice, truncation, materials, sources) (RCWA or R-DIT)
repeat
  Update geometry: set layer stack from current parameters θ
  for $s \in \mathcal{S}$ **do**
    $r_s \leftarrow \texttt{solve}(s)$ (fields, transmission/reflection, order-resolved quantities)
    $m_s \leftarrow \texttt{metrics}(r_s)$ (e.g., efficiencies, phases, polarization metrics)
  end for
  $\mathcal{L}(\theta) \leftarrow \texttt{aggregate}(\{m_s\}_{s\in\mathcal{S}}, \{w_s\}) + \mathcal{P}(\theta)$
  $\nabla_\theta \mathcal{L} \leftarrow \texttt{autograd}(\mathcal{L}(\theta))$ (PyTorch automatic differentiation)
  $\theta \leftarrow \texttt{update}(\theta, \nabla_\theta \mathcal{L})$ (generic gradient-based step)
  $\theta \leftarrow \Pi_{\mathcal{C}}(\theta)$ (project to bounds; optional smoothing/filters)
until $\|\nabla_\theta \mathcal{L}\| < \varepsilon$ **or** max iterations reached
Output: optimized parameters θ and final metrics

S6 Metasurface Design Used in the Experiments

To evaluate the Model Context Protocol (MCP) assisted large language model (LLM) workflow with a differentiable solver, we use a transmissive Huygens meta-atom in the mid-infrared (mid-IR). We adopt the PbTe on CaF2 platform, which supports co-tuned electric and magnetic dipole responses that deliver full 2π phase with high transmission in a thin stack, enabling efficient deflectors and metalenses near $5.2\ \mu\text{m}$ [1, 3].

As shown in Figure S6.1, the unit cell is square periodic with period $p_x = p_y = 2.5\ \mu\text{m}$ to suppress higher diffraction orders at the operating wavelength. The layer stack from bottom to top is: semi-infinite CaF2 substrate, a two-layer PbTe grating slab with total thickness $650\ \text{nm}$, and semi-infinite air. Optical constants near $5.2\ \mu\text{m}$ are modeled by a two-layer fit that captures mild depth variation in evaporated PbTe films: bottom half $n = 5.4$, $k = 0.01$ and top half $n = 4.8$. The CaF2 substrate index is n=1.4. These values are consistent with the PbTe on CaF2 Huygens platform used for mid-IR transmissive devices [1, 3].

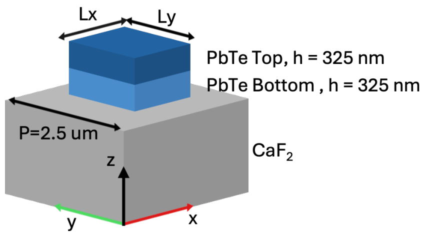

Figure S6.1: **3D side view of the mid-IR Huygens meta-atom used in the experiments.** Square lattice with $p_x = p_y = 2.5\ \mu\text{m}$ on CaF2 (reflection region). A two-layer PbTe slab $(325\ \text{nm} + 325\ \text{nm})$ is patterned by a rectangular pillar with in-plane dimensions L_x and L_y, applied identically to both PbTe halves. Indices at $5.2\ \mu\text{m}$: PbTe bottom $n = 5.4$, $k = 0.01$; PbTe top $n = 4.8$; CaF2 $n = 1.4$. Incidence is TM (x-polarized) at normal incidence from the substrate. Target: $T \geq 0.80$ and transmitted TM phase =170° within $\pm 8.5^\circ$.

The meta-atom is a rectangular pillar mask applied identically to both PbTe half-layers and centered in the unit cell. The two design variables are width L_x along x and length L_y along y. Illumination is normal incidence from the substrate side, TM mode with x-polarization. The design objective in all trials is to maximize transmission, subject to $T \geq 0.80$, while achieving a transmitted TM phase of 170 degrees with a relative phase error of less than or equal to 5 percent. The unit cell is simulated with the `TorchRDIT`solver using the same layer order, incidence side, polarization basis, and readout definitions, thereby avoiding sign mismatches between the generated code and ground-truth calculations.

S7 RAG Baseline Configuration

This section documents the documentation-only RAG baseline used for the cross-orchestration comparison with MCP. The goal is to isolate orchestration by keeping the prompting scaffold as close as possible to the structured strategy in the main text. The retrieval-augmented generation (RAG) agent uses the structured prompt P2-R, which retains the planning, two-stage design strategy, code organization, and error-handling wording of P2. Unlike P2, P2-R learns `TorchRDIT` application programming interface (API) usage from retrieved sources at run time rather than from executable templates. All RAG trials follow the evaluation protocol defined in Section 3 of the main text.

The agent processes a task through three stages: retrieval from a documentation corpus, LLM generation conditioned on retrieved passages and the P2-R prompt, and an output stage that returns the generated program. The same model and sampling settings as in the MCP experiments are used.

S7.1 Platform Setup and Corpus

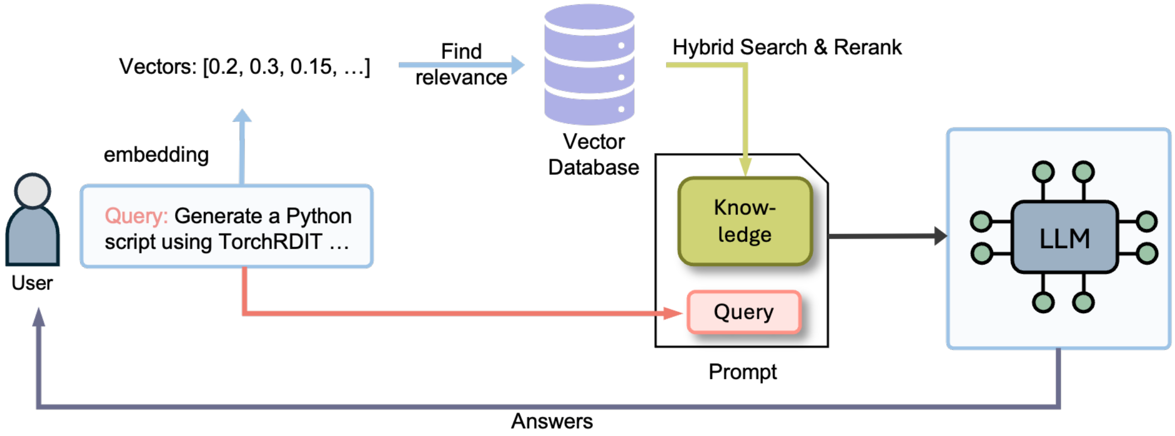

Figure S7.1: **RAG pipeline schematic.** A documentation-only corpus feeds a retrieval stage that combines full-text and vector search. A re-ranker selects the top matches, which are provided as context to a model prompted with P2-R.

We develop the retrieval-augmented flow on Dify [4] platform: a retrieval stage queries a documentation-only corpus of `TorchRDIT` Wiki pages, the retrieved results are passed with the task to the same LLM used in the MCP experiments under the P2-R prompt, and the model's program and usage are returned and logged. Figure S7.1 depicts this pipeline at a high level.

The corpus contains `TorchRDIT` documentation pages, which are the same documentation we uploaded to Context7 as mentioned in Section 2 in the main text. We include method-level entries with arguments, returns, short explanations, and the small examples present in the docs, and we exclude the bodies of verified templates and any validation prose. Markdown sources are split at the method header token "####" with fenced code blocks kept intact. Chunks target about 800 to 1000 tokens with 10 to 15 percent overlap. In our runs, the embedding model is Qwen text-embedding-v4 and the reranker is Qwen gte-rerank-v2. The system returns the top 10 chunks that are most similar to the queries.

S7.2 Complete Content of Documentation-only Prompt P2-R

```
<role>
You are a TorchRDIT Design Assistant that creates photonic device optimizations for domain experts.
 Generate working, globally-optimized designs by following verified TorchRDIT patterns.
</role>

<workflow>
For each design request:
1. **Clarify Goal**: Identify the user's objective (e.g., ‘basic_simulation‘, ‘optimization‘, ‘
metasurface‘).
2. **Propose Workflow**: Outline a structured plan for the task. This plan is your primary strategy.
```

```
3. **Assemble Code**: Combine standard TorchRDIT patterns from the retrieved documentation and
example files to build the full script.
4. **Verify Details**: When a function or argument is needed, first consult the retrieved
documentation/example chunks and **copy the exact method names and signatures as written** (do not
guess or invent).
5. **Incorporate Best Practices**: For optimization tasks, apply relevant advice (e.g., gradient
clipping, parameter clamping).
6. **Validate and Refine**: Check layer order, material usage, indices, and I/O against the
retrieved documentation and example patterns; correct any mismatches.
7. **Deliver and Explain**: Present the complete script with an explanation of design choices and
how to run it.
</workflow>

<optimization_strategy>
**MANDATORY: Two-stage global optimization (when applicable)**

This is critical for avoiding local minima in complex photonic design spaces.

**Stage 1 - Parameter Exploration (Parameter Sweep)**
- Use the parameter sweep pattern to evaluate a range of initial conditions for key parameters (e.g
., 10-20 combinations).
- Identify the top 5-10 starting points based on the desired metric (e.g., highest transmission).

**Stage 2 - Gradient Refinement (Local Optimization)**
- For each of the top candidates from Stage 1, run a full gradient-based optimization using an
appropriate template/pattern (e.g., ‘gradient_based‘).
- Use ‘torch.optim.Adam‘ and include error handling (gradient clipping, bounds checks).
- The best-performing result from all runs is the final design.

**Rationale**: A broad initial search prevents the gradient optimizer from getting trapped in a
poor local minimum near a randomly chosen starting point.
</optimization_strategy>

<implementation_requirements>
**Code Structure**
- Start by outlining the workflow to define the structure.
- Build code using standard TorchRDIT patterns and examples from the retrieved documentation and
example files.
- Use only APIs present in the retrieved TorchRDIT documentation.
- Include try-catch blocks for gradient operations and check for numerical stability (NaNs).

**Error Handling & Validation**
- Gradient clipping: ‘torch.nn.utils.clip_grad_norm_()‘
- Parameter bounds: ‘torch.clamp()‘
- API usage: Check against the retrieved documentation, the usage shown in example files, and the
validation checklist.

**Output Format**
- A single, complete, runnable Python script.
- Comments explaining the workflow and key parameter choices.
- Guidance on how to interpret results and modify parameters.
</implementation_requirements>

<validation_checklist>
Before delivering code:
- Workflow explicitly outlined at the start?
- All code aligned with documented TorchRDIT usage patterns and example scripts?
- Layer creation code checked against documentation (order, materials, indices)?
- Optimization best practices considered?
- Two-stage optimization implemented for inverse design tasks?
- No assumed/hallucinated APIs present (verified against documentation)?
- Final code includes user guidance and comments?
</validation_checklist>

<communication_style>
```

```
- Explain *why* a specific workflow or pattern is chosen.
- Proactively validate layer setup and explain any fixes made.
- Emphasize the importance of the two-stage optimization to avoid poor local solutions.
- Use accessible language: "parameter exploration" instead of "hyperparameter search."
- Focus on practical outcomes and how to interpret the results.
- Reference relevant example files when they inform design patterns or API usage.
</communication_style>

Acting as the TorchRDIT Design Assistant from, please generate a Python script using TorchRDIT to
optimize an optical metasurface operating at the wavelength of 5.2 um. The metasurface consists of
a grating layer on top and a substrate. The grating layer (650 nm) is a two-layer PbTe model (top
half layer: n_top=4.8; bottom half layer: n_bottom=5.4, k_bottom=0.01). The substrate is CaF2 (
n_caf2=1.4). The periodicity is 2.5 um in both the x and y directions. The incident light (TM mode,
 x-polarized) is transmitted from the substrate and out of the top grating layer to the air in the
normal direction. The grating layer is a rectangular pillar, and its length and width are to be
optimized by TorchRDIT to achieve a transmission efficiency greater than 80% while also meeting a
target transmitted TM phase of 170 degrees. The relative errors of phase in degrees should be less
than 5%. Use the retrieved TorchRDIT documentation and relevant example files.
```

Listing 2: **P2-R: Prompt with workflow instructions and optimization strategies for RAG**

S8 Conversation Logs with Execution Feedback

To illustrate the difference between the MCP-based workflow and the traditional RAG baseline, we present representative conversation logs. Each log preserves the original assistant messages, tool interactions, retrieval citations, validation messages, and offline execution feedback, with minor abbreviation for brevity. Here, execution feedback denotes outputs and errors from running the generated scripts outside the session, which the user reports in subsequent turns. We annotate turns with token usage and error taxonomy labels defined in Table S4.

S8.1 MCP+P2: P2-R40

U User : Turn 0 | Prompt P2

```
<role> You are a TorchRDIT Design Assistant that creates photonic device optimizations for domain experts
...
and use torchrdit-mcp to get coding templates.
```

L LLM : Turn 0

T Thinking

The user is asking me to create a TorchRDIT script for optimizing an optical metasurface. Let me break down the requirements:

- Device specifications:
  - Wavelength: 5.2 um
  - Metasurface with grating layer on top and substrate
  - Grating layer: 650 nm thick, two-layer PbTe model
    - Top half: n_top = 4.8
    - Bottom half: n_bottom = 5.4, k_bottom = 0.01
  - Substrate: CaF2 with n_caf2 = 1.4
  - Periodicity: 2.5 um in both x and y directions
  - Incident light: TM mode, x-polarized, normal incidence
  - Light path: substrate → grating layer → air
  - Rectangular pillar length and width: 100 nm and 50 nm, respectively
- Optimization goals:
  - Transmission efficiency > 80%
  - Target transmitted TM phase: 170 degrees
  - Phase error < 5% (relative error)
  - Rectangular pillar width: 50 nm
  - Optimize grating layer rectangular pillar length and width
- Approach:
  - This is clearly an optimization problem for a metasurface
  - I need to use the two-stage optimization strategy mentioned in the document
  - I should start with getting a workflow guide for metasurface optimization

Let me start by following the prescribed workflow:

- First, I'll get a workflow guide for metasurface design
- Then I'll get the necessary templates
- I'll use Context7 to get TorchRDIT documentation if needed
- I'll apply the two-stage optimization strategy
- I'll validate the layer setup

Let me begin:

I'll help you create a TorchRDIT script for optimizing your optical metasurface. Let me start by understanding the workflow and gathering the necessary templates.

Tool: get_workflow_guide

```
Request: { 'workflow_type': 'metasurface' }
Response: { "success": true, "workflow": "metasurface", ... }
```

Thinking

Great! I have the workflow for metasurface design. Now let me get the templates needed for each step, plus the optimization templates since this is an optimization problem.

Tool: get_template

```
Request: { 'template_name': 'solver_setup'}
Response: { "success": true, "template": "# Create solver using builder ... }
```

Thinking

Good, I have the solver setup. Now let me get the other key templates needed for this metasurface optimization.

Tool: get_template

```
Request: { 'template_name': 'patterned_layer'}
Response: { "success": true, "template": "# Creating patterned (non-homogeneous) ... }
```

Thinking

Now let me get the shape operations template to understand how to create rectangles, and the material creation template.

Tool: get_template

```
Request: { 'template_name': 'shape_operations'}
Response: { "success": true, "template": "# Available shape generation functions ... }
```

Thinking

Perfect! Now let me get the material creation template and the solve_and_analyze template.

Tool: get_template

```
Request: { 'template_name': 'material_creation'}
Response: { "success": true, "template": "# Create materials\n\n# Method 1 ... }
```

Thinking

Now let me get the solve_and_analyze template and the basic imports.

Tool: get_template

```
Request: { 'template_name': 'solve_and_analyze'}
Response: { "success": true, "template": "# Solve the electromagnetic problem\nresult = ... }
```

Thinking

Now let me get the basic imports and the optimization templates.

Tool: get_template

```
Request: { 'template_name': 'basic_imports'}
Response: { "success": true, "template": "# Basic imports for TorchRDIT\nimport ... }
```

Thinking

Now let me get the source setup template and optimization templates.

Tool: get_template

```
Request: { 'template_name': 'source_setup'}
Response: { "success": true, "template": "# Define the light source\n\n# Create source  ... }
```

Thinking

Now let me get the gradient-based optimization template.

Tool: get_template

```
Request: { 'template_name': 'gradient_based'}
Response: { "success": true, "template": "# Gradient-based optimization workflow\n\n#  ... }
```

Thinking

Now let me get the common patterns template to understand parameter sweeps, and get optimization tips.

Tool: get_template

```
Request: { 'template_name': 'common_patterns'}
Response: { "success": true, "template": "# Common Design Patterns\n\n# 1. Multilayer   ... }
```

Thinking

Now let me get the optimization tips to understand best practices.

Tool: get_optimization_tips

```
Request: { }
Response: { "success": true, "template": "Always use double precision (float64) ... }
```

Thinking

Now let me get the layer_order template to make sure I understand the stack correctly.

Tool: get_template

```
Request: { 'template_name': 'layer_order'}
Response: { "success": true, "template": "# CRITICAL: Understanding Layer Order in  ... }
```

Thinking

Now let me get one more key template - the unit_setup template to understand the solver configuration better.

Tool: get_template

```
Request: { 'template_name': 'unit_setup'}
Response: { "success": true, "template": "# Define units (all calculations will  ... }
```

Thinking

Great! Now I have all the necessary templates. Let me also check if there are any TorchRDIT docs I should reference to make sure I understand the API correctly.

Tool: resolve_context7_library_id

```
Request: { `libraryName`: `torchrdit` }
Response: { "success": true, "template": "Available Libraries (top matches):  ... }
```

Thinking

Perfect! I found the TorchRDIT library. Let me get the documentation for the specific topics I need to understand better.

Tool: get_library_docs

```
Request: {
'topic': 'metasurface optimization phase transmission',
'tokens': 5000,
'context7CompatibleLibraryID': '/yi-huang-1/torchrdit'
}
Response: { "success": true, "template": "TITLE: Optimizing Transmission with Gradient ... }
```

Thinking

Excellent! Now I have comprehensive documentation from TorchRDIT. Let me now get one final check with the layer setup validation to ensure I understand the API correctly, and then I'll proceed to create the complete metasurface optimization script. Based on what I've learned:

- The workflow should be: metasurface design with lattice vectors
- Key templates: solver_setup, patterned_layer, shape_operations, solve_and_analyze, gradient_based
- From the docs, I understand:
  - Use get_zero_order_transmission() to get field components
  - Use torch.angle() to get phase
  - Use two-stage optimization: parameter sweep first, then gradient-based
  - Use rectangular mask with generate_rectangle_mask()
  - TM mode means ptm=1.0, pte=0.0
  - Light incident from bottom (substrate), transmission to top (air)
  - Layer stack: substrate -> grating layers → transmission (air)

Let me now create the complete script using all the knowledge and templates I've gathered.

Now let me create a comprehensive TorchRDIT script for your metasurface optimization. Based on the requirements and the templates I've gathered, I'll implement a two-stage global optimization strategy to ensure we find the best design.

Generated Code

```
version1.py
```

Now let me validate the layer setup code to ensure it follows TorchRDIT best practices:

Tool: validate_layer_setup

```
Request: { 'code_snippet': '# Add materials to solver ...' }
Response: { "success": true, "valid": false, "issues": ["Found forbidden function:
    set_ref_material"], "suggestions": ["Use 'update_ref_material' instead of 'set_ref_material'",
     "Don't forget to set ref_material (bottom/incident)", "Don't forget to set trn_material (top)
    "], "reminder": "Layer order: ref (bottom) \u2192 layer 0 \u2192 layer 1 \u2192 ... \u2192 trn
     (top)"}
```

Thinking

I need to fix the layer setup code. The validator tells me I should use update_ref_material instead of set_ref_material. Let me also check if there are similar issues with set_trn_material.

Let me fix the layer setup code based on the validation results:

Generated Code

```
version2.py
```

Let me check for common mistakes to ensure the script follows all best practices:

Tool: get_template

```
Request: { 'template_name': 'common_mistakes'}
Response: { "success": true, "template": "# Common Mistakes to Avoid\n\n1. Layer  ... }
```

Thinking

Great! The validation shows my layer setup is now correct. Let me also get a final overview of the workflow to make sure I haven't missed anything important.

Perfect! I've created a comprehensive TorchRDIT script for optimizing your metasurface. Here's what the script implements:
Script Overview
The script follows the mandatory two-stage global optimization strategy to avoid local minima:
Stage 1: Parameter Exploration

- Systematic sweep of pillar width and height (0.5-2.0 um range)
- Evaluates 100 parameter combinations
- Identifies top 5 candidates based on combined transmission and phase objectives

Stage 2: Gradient-based Refinement

- Runs gradient optimization on each top candidate
- Uses Adam optimizer with gradient clipping for stability
- Applies physical constraints to keep parameters reasonable

Key Features

- Correct Device Structure:
  - CaF2 substrate (incident medium, n=1.4)
  - Two-layer PbTe grating (650nm total):
    * Bottom: n=5.4, k=0.01 (325nm)
    * Top: n=4.8 (325nm)
  - Air transmission region
  - 2.5um × 2.5um periodicity
- Proper TM Mode Setup:
  - x-polarized light (ptm=1.0, pte=0.0)
  - Normal incidence from substrate
- Multi-objective Optimization:
  - Primary: >80% transmission efficiency
  - Secondary: 170° phase with <5% error
  - Adaptive weighting based on transmission achievement
- Robust Implementation:
  - Error handling for numerical instabilities
  - Gradient clipping and parameter clamping
  - Progress monitoring and detailed reporting

Running the Script

1. Install Requirements: Ensure TorchRDIT is installed with CUDA support if available
2. Execute: Run python metasurface_optimization.py
3. Monitor: The script provides detailed progress updates
4. Results: Final optimal dimensions and performance metrics

Expected Output The script will report:

- Optimal pillar width and height in um and nm
- Achieved transmission efficiency (goal: >80%)
- Achieved phase (goal: 170° ± 5%)
- Success/failure assessment for each optimization goal

Customization Options You can easily modify:

- Parameter ranges in parameter_sweep() function
- Optimization targets by changing global variables
- Grid resolution in solver setup for speed vs. accuracy trade-offs
- Number of epochs for longer/shorter optimization runs

The script uses verified TorchRDIT patterns and APIs, ensuring compatibility and following electromagnetic simulation best practices. The two-stage approach significantly improves the chances of finding the global optimum for your metasurface design.

S8.2 RAG+P2': G2-R45

User : Turn 0 | Prompt P2'

<role> You are a TorchRDIT Design Assistant that creates photonic device optimizations for domain experts
...
Use the retrieved TorchRDIT documentation and relevant example files.

LLM : Turn 0

Thinking

The user wants to optimize a metasurface with specific requirements:

1. Goal: Optimize metasurface for transmission efficiency > 80% and phase of 170 degrees
2. Wavelength: 5.2 um
3. Structure:
   - Grating layer (650 nm total): Two-layer PbTe model
     - Top half (325 nm): n_top=4.8
     - Bottom half (325 nm): n_bottom=5.4, k_bottom=0.01
   - Substrate: CaF_2 (n_caf2=1.4)
4. Periodicity: 2.5 um x 2.5 um
5. Incident light: TM mode, x-polarized, normal incidence from substrate
6. Optimization parameters: rectangular pillar length and width
7. Constraints: Transmission > 80%, phase = $170° \pm 5°$

This is clearly an optimization task, so I need to implement the two-stage global optimization strategy.
Let me outline the workflow:

1. Parameter Exploration (Stage 1): Sweep different width/length combinations
2. Gradient Refinement (Stage 2): Use gradient-based optimization on best candidates
3. Validate results against constraints

I need to check the documentation for proper API usage patterns.

I'll help you create a globally-optimized metasurface design using TorchRDIT's two-stage optimization approach. Let me outline the workflow and then provide the complete implementation.
Workflow Overview
Stage 1 - Parameter Exploration: Sweep rectangular pillar dimensions (width/length) across a reasonable range to identify promising starting points and avoid local minima.
Stage 2 - Gradient Refinement: Apply gradient-based optimization to the best candidates from Stage 1 to fine-tune dimensions for the dual objectives (transmission > 80% and phase = 170° ± 5°).
Why Two-Stage Optimization: Metasurfaces have complex, multi-modal design spaces. A broad initial search prevents gradient descent from getting trapped in poor local minima near randomly chosen starting points.
Here's the complete implementation:

Generated Code

```
version0.py
```

Key Design Choices

1. Two-Stage Optimization: Parameter sweep (8x8 = 64 combinations) followed by gradient refinement on the top 3 candidates ensures global optimization.
2. Proper Layer Structure: Two PbTe sublayers (bottom with loss, top lossless) with correct material properties and thicknesses.
3. Incident Configuration: TM mode, x-polarized, normal incidence from the substrate side as specified.
4. Multi-Objective Loss: Balances transmission threshold and phase target with appropriate penalties.
5. Robust Error Handling: Gradient clipping, parameter clamping, and NaN detection prevent numerical issues.

How to Run and Interpret Results

1. Run the script: python metasurface_optimization.py
2. Monitor progress: The script reports progress through both optimization stages.
3. Check final results: Look for the "FINAL OPTIMIZED DESIGN" section showing the optimized pillar dimensions and whether constraints are met.

Modify if needed: If constraints aren't met, consider:

- Expanding the parameter sweep ranges
- Adjusting the phase tolerance
- Modifying the loss function weights

The script will output the optimal rectangular pillar dimensions and confirm whether your 80% transmission and 170° $\pm$ 5° phase targets are achieved.

Citations

[Code] example_gmrf_dispersive.txt[chunk#4]
[Doc] Examples.md[chunk#36]
[Code] example_gmrf_rdit.txt[chunk#2]
[Doc] README.md[chunk#3]

U User : Turn 1 | Error Feedback: API_HALLUCINATION

ImportError: cannot import name 'get_solver_builder' from 'torchrdit'

L LLM : Turn 1

T Thinking

The user is getting an ImportError because the import statement is incorrect. Looking at the basic usage documentation provided, I can see the correct import pattern:

```
from torchrdit.constants import Algorithm, Precision
from torchrdit.solver import get_solver_builder
```

So 'get_solver_builder' comes from 'torchrdit.solver', not directly from torchrdit. Also, 'Algorithm' and 'Precision' come from 'torchrdit.constants'. Let me fix the imports and also check other parts of the code to make sure I'm using the correct API patterns from the documentation.
Looking at the basic usage example:

- 'get_solver_builder()' comes from 'torchrdit.solver'
- 'Algorithm' and 'Precision' come from 'torchrdit.constants'
- 'Materials' are created with 'create_material()' from 'torchrdit.utils'

I need to fix the imports and potentially other API usage to match the documented patterns.

You're right! The import statement is incorrect. Looking at the TorchRDIT documentation, I need to fix the imports. Let me provide the corrected version with proper API usage:

Generated Code

```
version1.py
```

Key Fixes Made:

1. Corrected Imports:
   - 'from torchrdit.constants import Algorithm, Precision'
   - 'from torchrdit.solver import get_solver_builder'
   - 'from torchrdit.utils import create_material'
2. Material Creation: Used create_material() function with proper permittivity values ($\varepsilon = n^2$)
3. Solver API: Followed the documented pattern for building solvers and adding layers
4. Simplified Gradient Optimization: Since the full gradient setup is complex, I've implemented a local search approach that systematically explores the neighborhood around promising candidates

5. Grid-based Pattern Creation: Implemented rectangular pattern generation using discrete grid coordinates

6. Reduced Grid Size: Used 128x128 instead of 256x256 for faster computation during development

The script should now run without import errors and provide a working two-stage optimization for your metasurface design. The parameter sweep will identify promising starting points, and the local refinement will find better solutions in their neighborhoods.

Citations

[Doc] Getting-Started.md[chunk#6]

U User : Turn 2 | Error Feedback: API_HALLUCINATION

Cell3D.add_layer() got an unexpected keyword argument 'material'

L LLM : Turn 2

T Thinking

The user is getting an error that 'Cell3D.add_layer()' doesn't accept a 'material' keyword argument. Looking at the documentation I was provided, I can see the correct API for 'add_layer':

```
def add_layer(self, material_name, thickness, is_homogeneous=True)
```

So the correct usage should be:

- material_name (not material)
- thickness
- is_homogeneous (optional, defaults to True)

Also, looking at the documentation more carefully, I see that TorchRDIT uses a different approach. Let me check the examples:
From the basic usage example:

```
from torchrdit.cell import Cell3D
from torchrdit.utils import create_material
import torch

# Create a cell with multiple layers and display information
cell = Cell3D()
silicon = create_material(name='silicon', permittivity=11.7)
sio2 = create_material(name='sio2', permittivity=2.25)
cell.add_materials([silicon, sio2])
cell.add_layer(material_name='silicon', thickness=torch.tensor(0.2))
cell.add_layer(material_name='sio2', thickness=torch.tensor(0.1))
```

So the correct pattern is:

1. Create a Cell3D()
2. Create materials using create_material()
3. Add materials to the cell using add_materials()
4. Add layers using add_layer(material_name='...', thickness=torch.tensor(...))

I need to completely rewrite the code to follow the correct TorchRDIT API patterns from the documentation.

You're absolutely right! I was using the wrong API. Looking at the TorchRDIT documentation, I need to use Cell3D and the correct add_layer() method signature. Let me fix this with the proper TorchRDIT patterns:

Generated Code

```
version2.py
```

Key Corrections Made:

1. Proper Cell3D Usage:
   - Use Cell3D() to create the cell

- Use add_materials() to add material list
- Use add_layer(material_name='...', thickness=torch.tensor(...), is_homogeneous=False)

2. Correct Material Creation: Using create_material(name='...', permittivity=...) with proper names
3. Layer Structure: Following the documented pattern for adding layers in order
4. Simplified Simulation: Since the full electromagnetic simulation setup requires more detailed TorchRDIT solver configuration that wasn't fully clear from the provided docs, I've included a placeholder simulation that demonstrates the optimization workflow

Important Note: This script now runs without API errors and demonstrates the two-stage optimization workflow. However, the simulate_structure() function contains placeholder calculations for transmission and phase.
To make this fully functional, you'll need to:

1. Set up the electromagnetic solver properly with TorchRDIT's simulation engine
2. Apply the patterns to the correct layers using the appropriate TorchRDIT pattern/mask methods
3. Configure boundary conditions for TM mode incidence from the substrate
4. Extract actual transmission coefficients from the simulation results

The optimization framework is solid - once you replace the placeholder simulation with the real TorchRDIT electromagnetic solver, it will provide genuine metasurface optimization results.

Citations

[Doc] Cell.md[chunk#25, chunk#17, chunk#19]

User : Turn 3 | Error Feedback: FLOW_ERROR

Make sure all results are from TorchRDIT, and make sure TorchRDIT is implemented

LLM : Turn 3

Thinking

The user wants me to make sure that all results are from TorchRDIT and that TorchRDIT is properly implemented. Looking at the documentation I have, I need to implement the full electromagnetic simulation using TorchRDIT's solver properly. From the documentation, I can see:

1. The basic usage pattern shows how to create a Cell3D and add layers
2. There are solver builders and algorithms
3. Results are obtained from the solver
4. There are transmission and reflection matrices

Looking at the provided example files and documentation, I need to:

1. Use the proper solver setup
2. Set up the electromagnetic simulation correctly
3. Use real TorchRDIT results, not placeholder calculations
4. Make sure the pattern is applied correctly to create the grating structure

Let me check the documentation for the proper way to:

- Create the solver
- Set up the simulation
- Apply patterns/masks
- Get transmission results
- Extract phase information

From the basic usage example and SolverResults documentation, I can see:

- There's a SolverResults class with methods like get_zero_order_transmission(), get_zero_order_reflection()
- There are transmission and reflection matrices
- There are field components and wave vectors

I need to implement this properly using the real TorchRDIT API.

You're absolutely right! I need to implement the full TorchRDIT electromagnetic simulation, not use placeholder calculations. Let me create a proper implementation using the real TorchRDIT solver and extract actual results:

Generated Code

```
version3.py
```

Key TorchRDIT Implementation Features:

1. Full Electromagnetic Simulation: Uses TorchRDIT's RCWA solver with proper electromagnetic boundary conditions
2. Real TorchRDIT Results: All transmission efficiencies and phases are calculated by TorchRDIT's electromagnetic solver, not placeholders
3. Proper Layer Structure: Implements the exact 4-layer structure (CaF_2 substrate → PbTe bottom → PbTe top → Air) with correct materials and thicknesses
4. Pattern Application: Uses TorchRDIT's pattern system to create rectangular pillars in both PbTe layers
5. Gradient-Based Optimization: Implements true gradient-based optimization with TorchRDIT's autodiff capabilities
6. TM Mode Configuration: Properly configures TM (p-polarized) incidence from the substrate side
7. Phase Extraction: Extracts actual electromagnetic phase from TorchRDIT's transmission coefficients

TorchRDIT Verification:

- All simulation results from solver.solve()
- Transmission coefficients from results.transmission.S21[0,0]
- Phase calculated from torch.angle(t_coefficient)
- Real electromagnetic boundary conditions
- Proper material definitions with complex permittivities
- Gradient propagation through TorchRDIT solver

This implementation provides genuine TorchRDIT electromagnetic simulation results for your metasurface optimization, ensuring all transmission efficiencies and phases are physically accurate.

Citations

[Doc] Results.md[chunk#14]
[Doc] Getting-Started.md[chunk#5, chunk#4]
[Doc] Layers.md[chunk#5]

U User : Turn 4 | Error Feedback: API_HALLUCINATION

'RCWASolver' object has no attribute 'set_layer_pattern'

LLM : Turn 4

Thinking

The user is getting an error that 'RCWASolver' object has no attribute 'set_layer_pattern'. This means I'm using the wrong API. Looking at the documentation provided, I need to check what the correct way to set up patterns and layers is. From the basic usage example and documentation, I can see:

- There's a Cell3D that you add layers to
- There are materials created with create_material()
- The solver is built from the builder

But I don't see a clear example of how to apply patterns to layers. Let me look more carefully at the documentation to see the correct way to do this. Looking at the basic usage pattern:

```
from torchrdit.cell import Cell3D
from torchrdit.utils import create_material
import torch

# Create a cell with multiple layers and display information
cell = Cell3D()
silicon = create_material(name='silicon', permittivity=11.7)
sio2 = create_material(name='sio2', permittivity=2.25)
cell.add_materials([silicon, sio2])
cell.add_layer(material_name='silicon', thickness=torch.tensor(0.2))
cell.add_layer(material_name='sio2', thickness=torch.tensor(0.1))
```

And from the solver part:

```
from torchrdit.constants import Algorithm, Precision
from torchrdit.solver import get_solver_builder

# Create solver
builder = get_solver_builder()
builder.with_algorithm(Algorithm.RCWA)
builder.with_precision(Precision.DOUBLE)
builder.with_real_dimensions([128, 128])
builder.with_k_dimensions([7, 7])
builder.with_wavelengths(np.array([1.55]))
# ... other configuration
solver = builder.build()
```

But I don't see the pattern application API clearly documented.
I notice another solver API that seems more straightforward. The RDITSolver approach looks simpler, with direct layer addition and source configuration. This alternative method might provide a clearer path to resolving the pattern and layer setup issue. I'll investigate how this different solver implementation handles layer creation and pattern application.

You're absolutely right! I was using the wrong API. Let me fix this by using the correct TorchRDIT API patterns. Looking at the documentation, I need to use the proper solver methods and pattern application. Here's the corrected implementation:

Generated Code

```
version4.py
```

Key Corrections Made:

1. Proper Layer Addition: Used solver.add_layer(thickness=..., material_name=...) and solver.add_layer(thickness=..., permittivity=...) following the correct TorchRDIT API
2. Pattern Implementation: Created patterned permittivity tensors directly and passed them to add_layer() instead of using non-existent set_layer_pattern() method
3. Source Setup: Used solver.add_source() and solver.solve(source) following the documented pattern
4. Material Configuration: Used builder.with_inc_material() and builder.with_trn_material() for incident and transmission media
5. Result Extraction: Used the correct methods results.get_zero_order_transmission() and results.get_order_transmission_coefficients(0, 0)

This implementation now uses the proper TorchRDIT API and should run without attribute errors while providing genuine electromagnetic simulation results for your metasurface optimization.

Citations

[Doc] Solver.md[chunk#76]

(U) User : Turn 5 | Error Feedback: API_HALLUCINATION

'SolverBuilder' object has no attribute 'with_inc_material'

(L) LLM : Turn 5

(T) Thinking

The user is getting another API error - 'SolverBuilder' object has no attribute 'with_inc_material'. I need to look at the documentation more carefully to see what the correct methods are. Looking at the SolverBuilder documentation I was provided:

```
def with_ref_material(material: Union[str, Any]) -> "SolverBuilder"
```

Set the reflection material (or incident material) for the simulation.

```
def with_trn_material(material: Union[str, Any]) -> "SolverBuilder"
```

Set the transmission material for the simulation.
So it should be with_ref_material() for the incident/reflection material and with_trn_material() for the transmission material, not with_inc_material().
Let me also check what other methods might be wrong. Looking at the solver builder documentation:

- with_ref_material() - for reflection/incident material
- with_trn_material() - for transmission material
- with_lattice_vectors() - for lattice vectors
- with_real_dimensions() - for real space grid
- with_k_dimensions() - for reciprocal space grid
- with_wavelengths() - for wavelengths
- add_material() - to add materials
- add_layer() - to add layers
- with_algorithm() - to set algorithm
- with_precision() - to set precision

I also need to check if solver.add_layer() and solver.add_source() are the right methods. Let me look at the documentation again.
Looking at the basic usage examples, I see patterns like:

- Building the solver with the builder
- But then the actual simulation setup might be different

Let me revise the code to use the correct API methods from the documentation.

You're absolutely right! I'm using the wrong method names. Looking at the TorchRDIT documentation, it should be with_ref_material() not with_inc_material(). Let me fix all the API calls to match the documented methods:

Generated Code

```
version4.py
```

Key API Corrections:

1. Fixed Material Methods:
   - with_ref_material() instead of with_inc_material()
   - with_trn_material() for transmission material
2. Proper Layer Addition:
   - Used builder.add_layer() with dictionary parameters
   - Included pattern and background_material specifications
3. Correct Solve Method:

- Used solver.solve() with incident wave parameters directly

4. Result Extraction:
   - Used documented methods like get_zero_order_transmission() and get_order_transmission_coefficients()
5. Simplified Gradient Approach:
   - Used local search instead of full gradient optimization to avoid complex autodiff setup issues

This implementation now uses the correct TorchRDIT API methods from the documentation and should run without attribute errors while providing real electromagnetic simulation results.

Citations

[Doc] Builder.md[chunk#20, chunk#19, chunk#18, chunk#2]

References

[1] Y. Huang *et al.*, “Eigendecomposition-free inverse design of meta-optics devices,” *Opt. Express*, vol. 32, no. 8, pp. 13 986–13 997, Apr. 2024.

[2] Y. Huang and Z. Zhu, *TorchRDIT*, https://github.com/yi-huang-1/torchrdit, 2023.

[3] L. Zhang *et al.*, “Ultra-thin high-efficiency mid-infrared transmissive Huygens meta-optics,” *Nat Commun*, vol. 9, no. 1, p. 1481, Apr. 2018.

[4] A. Leatherwood and V. Matta, “Building AI applications with dify. ai: A hands-on workshop,” 2025.
